# Ontogeny of the Middle-Ear Air-Sinus System in *Alligator mississippiensis* (Archosauria: Crocodylia)

**DOI:** 10.1371/journal.pone.0137060

**Published:** 2015-09-23

**Authors:** David L. Dufeau, Lawrence M. Witmer

**Affiliations:** 1 Department of Biological Sciences, Ohio University, Athens, Ohio, United States of America; 2 Department of Biomedical Sciences, Heritage College of Osteopathic Medicine, Ohio University, Athens, Ohio, United States of America; New York Institute of Technology College of Osteopathic Medicine, UNITED STATES

## Abstract

Modern crocodylians, including *Alligator mississippiensis*, have a greatly elaborated system of pneumatic sinuses invading the cranium. These sinuses invade nearly all the bones of the chondrocranium and several bony elements of the splanchnocranium, but patterns of postnatal paratympanic sinus development are poorly understood and documented. Much of crocodylomorph—indeed archosaurian—evolution is characterized by the evolution of various paratympanic air sinuses, the homologies of which are poorly understood due in large part to the fact that individual sinuses tend to become confluent in adults, obscuring underlying patterns. This study seeks to explore the ontogeny of these sinuses primarily to clarify the anatomical relations of the individual sinuses before they become confluent and thus to provide the foundation for later studies testing hypotheses of homology across extant and extinct Archosauria. Ontogeny was assessed using computed tomography in a sample of 13 specimens covering an almost 19-fold increase in head size. The paratympanic sinus system comprises two major inflations of evaginated pharyngeal epithelium: the pharyngotympanic sinus, which communicates with the pharynx via the lateral (true) Eustachian tubes and forms the cavum tympanicum proprium, and the median pharyngeal sinus, which communicates with the pharynx via the median pharyngeal tube. Each of these primary inflations gives rise to a number of secondary inflations that further invade the bones of the skull. The primary sinuses and secondary diverticula are well developed in perinatal individuals of *Alligator*, but during ontogeny the number and relative volumes of the secondary diverticula are reduced. In addition to describing the morphological ontogeny of this sinus system, we provide some preliminary exploratory analyses of sinus function and allometry, rejecting the hypothesis that changes in the volume of the paratympanic sinuses are simply an allometric function of braincase volume, but instead support the hypothesis that these changes may be a function of the acoustic properties of the middle ear.

## Introduction

**Fig 1:**
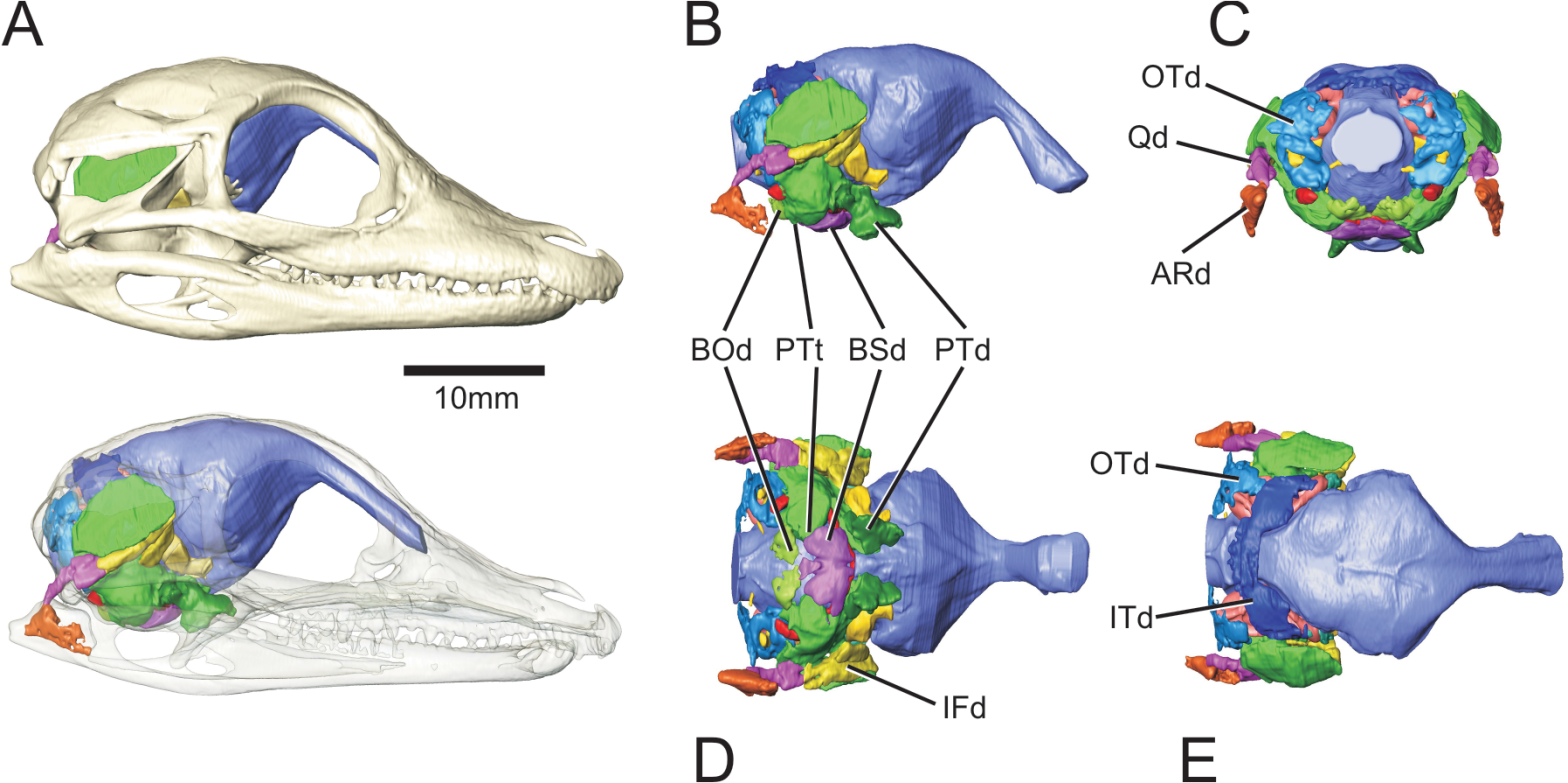
Full suite of paratympanic sinuses and diverticula in a perinatal *Alligator mississippiensis* (OUVC 10606). A. Rendered with skull and transparent skull. B. Left lateral view with brain endocast rendered in blue. C. Caudal view. D. Ventral view. E. Dorsal view. **ARd**, articular diverticulum; **BOd,** basioccipital diverticulum; **BSd**, basisphenoid diverticulum; **IFd**, infundibular diverticulum; **ITd**, intertympanic diverticulum; **OTd**, otoccipital diverticulum; **PTd**, pterygoid diverticulum; **PTt**, pharyngotympanic tube; **Qd**, quadrate diverticulum.

Modern crocodylians have a remarkable proportion of their skulls occupied by large recesses (Fig 1, S1 File, 3D pdf of perinatal Alligator mississippiensis). These recesses, which infiltrate every skeletal element of the braincase, have been the subject of nearly two hundred years of interpretation regarding their form, function, and evolutionary trajectory [1–6]. Despite this scrutiny, our understanding of these aspects of crocodylian head anatomy is still rudimentary, due in part to the nature and scope of previous investigations and to the limitations of earlier methods for discovering and reconstructing the form and extent of these recesses.

Cuvier [1] was the first to investigate the passages leading to these bony recesses, and in particular described a median foramen in the crocodile leading into a canal which ascends to the “sphenoid” and bifurcates at the “sella turcica.” He incorrectly interpreted these bifurcated ‘branches’ as opening into the hypophyseal fossa, and accordingly interpreted the structure to be an arterial foramen: “le trou des artères.” Owen [2] observed that the median foramen, along with two foramina situated laterally to it (the true Eustachian canals), conduct the epithelium of the pharynx into the skull, where it becomes continuous with the epithelium of the tympanic cavity. The nature of these braincase recesses, being evaginated pharyngeal epithelium that pneumatizes the skull and participate in the conformation of the middle ear, is well documented [3,5, 7–9].

**Fig 2:**
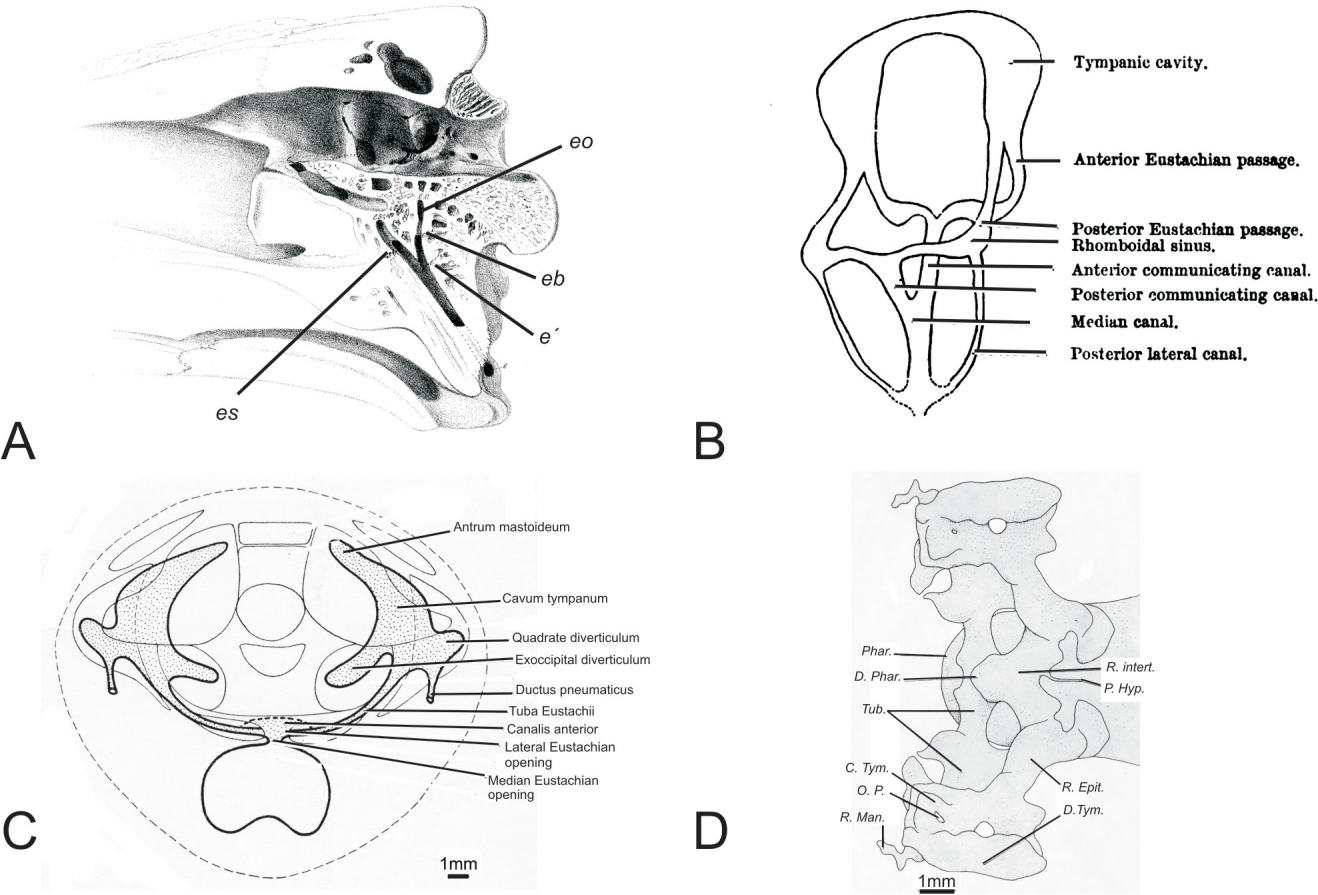
Previous representations of the crocodilian middle-ear space shown as A) a lithographic rendering of a parasagittal cut through the skull of a crocodile, adapted from Owen, 1850; B) a schematic depiction of the communicating canals of the tympanic cavity in rostral oblique view, adapted from Miall, 1878; C) a schematic showing the development of the tympanic cavity in a late stage embryo of *Crocodilus cataphractus* in rostrocaudal aspect, adapted from Müller, 1967; D) A schematic of the development of the tympanic cavity in a late stage embryo of *Alligator mississippiensis* in dorsal view, adapted from Simonetta, 1956. **C. Tym.,** Tympanic cavity; **D. Phar.,** Posterior pharyngeal diverticulum; **D. Tym.,** Tympanic diverticulum; **e’,** bifurcation of the median Eustachian canal; **eb,** bifurcation of the basioccipital branch, **eo,** basioccipital branch of the Eustachian canal; **es,** basisphenoidal branch of the Eustachian canal; **O.P.,** paratympanic organ; **Phar.,** Pharynx; **P. Hyp.,** Hypophyseal pedicle; **R. Epit.,** epitubaric recess; **R. Intert.,** intertympanic recess; **R. Man.,** mandibular recess; **Tub.,** Eustachian tube.

Although foundational, these works did not adequately address the extent, conformation and associations between the component recesses. Van Beneden [4: p. 559] was first to recognize in crocodylians 'a tendency [toward] well marked secondary diverticula formation of the cavity of the middle ear,' and also suggested the potential for confluence between intersecting diverticula. Nevertheless, the pattern of diverticular expansion was not explicitly demonstrated in van Beneden’s seminal work. Subsequent developmental work on late-stage embryos of alligator [6] and crocodile (*Mecistops cataphractus*) [10] show some of the early stages of diverticular development, but owing to limitations of serial histological sectioning (e.g., small sample sizes due to high labor, missing or damaged sections, difficulty reconciling spatial relationships beyond the plane of the sections), and to the limited ontogenetic scope of these works, trends in diverticular expansion and intersections were incompletely surveyed (Fig 2).

The paleontological community has also been interested in patterns of paratympanic pneumaticity and their ontogenetic development. Indeed, Colbert’s [5] research into the middle-ear structure of crocodylians was precipitated by his work on an extinct crocodyliform (e.g., *Sebecus icaeorhinus*) [11]. Likewise, Whetstone and Martin [12], Crompton and Smith [13], Tarsitano [14], Walker [15], Clark [16], and Brochu [17] (see also Rowe et al.)[18] all made important contributions in the study of braincase, middle-ear, and pneumatic structure in extant crocodylians as a means to better understand the morphology and evolution of extinct crocodylomorphs and other archosaurs. Indeed, the present study falls in this same category [19–21]. A common thread running through the paleontological studies is how the form and ontogeny of extant crocodylian middle-ear pneumatic diverticula can shed light on the diversity of pneumatic structures seen in the fossil record.

Although this study is concerned primarily with anatomical and ontogenetic analysis of the pneumatic diverticula, we also recognize that there are potential functional implications of these ontogenetic changes. The middle-ear space, including all its various diverticula, have direct functional bearing on auditory function in that the volume of the air space effects the impedance-matching function of the middle ear, such that larger volumes reduce the stiffness of the tympanic cavity, enhancing low-frequency hearing [22–24]. Furthermore, Wever and Vernon [25] showed that sound energy is conducted through the heads of crocodylians via intertympanic communications allowing for sound localization by way of the detection of interaural time and phase differences. More recent studies [26–28] presented the circuitry within the brainstem nuclei for calculating the interaural time differences and the biophysics of directional hearing. And although at least one study [12] compared the “periotic pneumatic cavities” of alligators to mammalian auditory bullae as they affect air compliance as related to transduction, none have looked explicitly at the acoustic performance of the middle-ear space in crocodylians. This study seeks to follow on these previous works, drawing on quantitative estimates of paratympanic diverticular volume and conformation through ontogeny to calculate the acoustic resonant properties of the middle ear that may have bearing on acoustic performance and auditory behaviors. We regard these as preliminary analyses that merit future experimental validation, but, as is discussed below, correlations with behavioral data from the literature are promising.

## Materials and Methods

### Ontogenetic sample

Salvage specimens were provided to this study by the Rockefeller Wildlife Refuge, Grand Chenier, Louisiana. Additional skeletal specimens were provided by the Field Museum of Natural History, Chicago, Illinois, and the National Museum of Natural History, Smithsonian Institution, Washington, D.C. An ontogenetic sample of 13 *Alligator mississippiensis* were examined to determine the relationships and extent of the paratympanic sinuses and diverticula (Table 1). No live animals were used in the design or execution of this study, and no animals were euthanized exclusively for use in this study. Specimens were chosen to represent the full range of postnatal ontogenetic stages, ranging from neonates measuring 29.3 mm dorsal skull length (DSL, measured from the caudal edge of the supraoccipital to the rostral tip of the premaxilla) to individuals approaching the asymptote of logistic growth (551.6 mm DSL). Juvenile and perinatal specimens (OUVC 10385–10391, OUVC 10117, OUVC 10606) were acquired from the Rockefeller Wildlife Refuge. The majority of these specimens were intact formalin-fixed or frozen heads. Larger specimens (OUVC 9761, OUVC 10629, USNM 211232, USNM 211233) were a mix of frozen heads and skeletal preparations (i.e., dried skulls). The use of an ontogenetic series allowed us to track the sequence of appearance and growth trajectories of expansions of the paratympanic sinus system throughout the skull.

**Table 1:** List of *Alligator mississippiensis* specimens used in current study.

Specimen	Skull length^1^ (mm)	Specimen type	Scan parameters
OUVC 10606	29.26	Fixed	45μm/slice 80kV@450μA
OUVC 10117	29.96	Skeletal	45μm/slice 80kV@450μA
OUVC 10385	46.1	Frozen	92μm/slice 60kV@450μA
OUVC 10386	56.9	Frozen	92μm/slice 60kV@450μA
OUVC 10387	57.6	Frozen	92μm/slice 60kV@450μA
OUVC 10388	61.2	Frozen	92μm/slice 60kV@450μA
OUVC 10389	84.1	Frozen	92μm/slice 60kV@450μA
OUVC 10390	85.4	Frozen	92μm/slice 60kV@450μA
OUVC 10391	87.1	Frozen	92μm/slice 60kV@450μA
OUVC 10629	185.8	Frozen	625μm/slice 120kV@200–300μA
OUVC 9761	301.5	Frozen	625μm/slice 120kV@200–300μA
USNM 211232	477.6	Skeletal	625μm/slice 120kV@200–300μA
USNM 211233	551.6	Skeletal	625μm/slice 120kV@200–300μA

^1^ Measured from the caudal edge of the supraoccipital to the rostral tip of the premaxilla.

### Determining sinus identity

Criteria for identifying paratympanic sinuses needed to be established before exploring patterns of sinus expression. Using gross dissection, we were able to identify paratympanic sinuses as (1) pharyngeally derived epithelial evaginations that (2) maintain a communication with the pharynx and (3) are pneumatically inflated (i.e., filled with air). Further, we were able to identify secondary epithelial diverticula derived from these sinuses that (1) excavate smooth bony recesses into the bones of the skull, and (2) maintain communication with the parent sinus via one or more ostia that often coincide with bony foramina. There are circumstances where the criteria listed above do not adequately delimit individual sinuses or diverticula, particularly where broad communications between adjacent sinuses or diverticula occur. In such cases, boundaries between adjacent diverticula are identified on the basis of well-defined constrictions in the excavated bony recesses, formed where two pneumatically inflated bubbles meet, similar in conformation to conjoined soap bubbles. These criteria and osteological correlates (i.e., smooth recesses and ostia) were subsequently translated to digital reconstruction methods (see below).

### Digital data acquisition

The majority of the sinus identification criteria outlined above can be observed radiographically (the epithelial sac that actually comprises the sinus is the exception, because surrounding denser tissues typically attenuate this soft-tissue signal below the range of recoverable values). The use of computed tomographic (CT) scanning has already been demonstrated to be an indispensable method for recovering three-dimensional (3D) morphological data on the paratympanic sinus system of crocodylians [19]. Accordingly, we CT-scanned our ontogenetic sample at sufficiently high resolutions (45, 92, and 625 μm slice thicknesses, depending on size of the head; see Table 1) to discern modalities conforming to air, soft tissue, and bone, and resolving fine-scale structures such as the columella, tympanum, and neurovascular canals. Juvenile specimens were scanned at the Ohio University MicroCT Facility, using a GE eXplore Locus *in vivo* Small Animal MicroCT Scanner. Images were acquired at scan resolutions of 45 and 92 μm using scan energies of 60kV at 450μA. The resulting data volume (in VFF format) was exported from GE Healthcare's MicroView 2.1.2 (http://sourceforge.net/projects/microview/) to the DICOM format. Larger specimens were scanned at OhioHealth O'Bleness Hospital, Athens, Ohio, using a GE LightSpeed Ultra MultiSlice CT scanner. These scans were acquired at a resolution of 625 μm using scan energies of 120kV and usually 200–300 mA. Data were output in the DICOM format.

### Digital data analysis and visualization

DICOM data were imported into Amira 4.1 (Mercury-TGS, Chelmsford, MA) on a 32-bit PC workstation running Debian 4.0 Linux (2.6.18 kernel) and equipped with an nVidia Quadro FX 3000 video card and 4 GB of RAM. Relevant anatomical features (e.g., sinuses) were identified and delimited in Amira using the criteria outlined above. The process of delimiting these spaces (facilitated by the “segmentation editor” tool in Amira) results in an anatomical selection or compartment that can be rendered in 3D separately from or together with other structures. Amira was also used to quantify measurements of sinus surface area and volume.

### Allometric analysis

**Fig 3:**
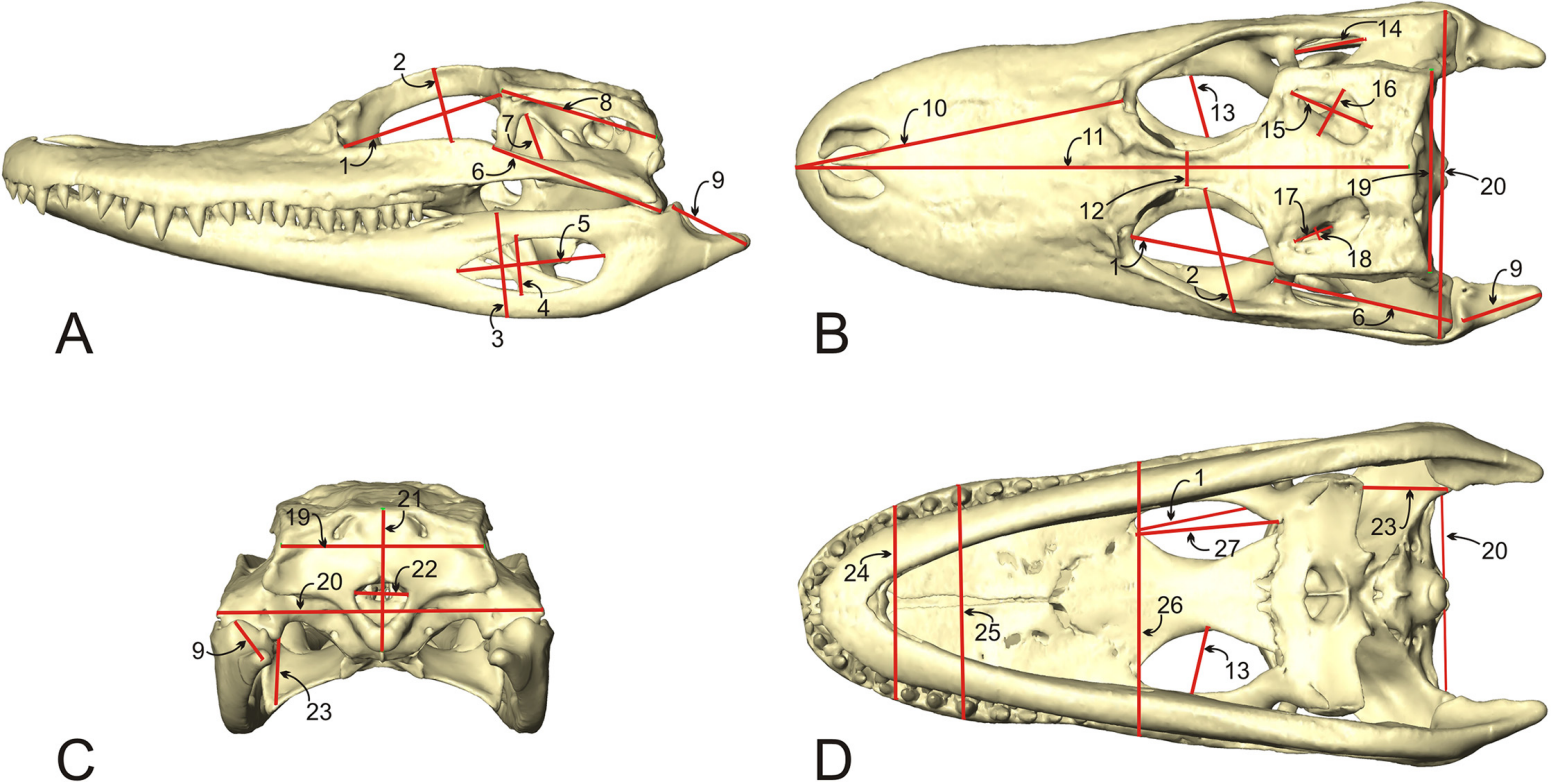
Skull measurements used to calculate Geometric Mean of skull size in *Alligator mississippiensis*, shown in A) left lateral, B) dorsal, C) caudal, and D) ventral views. Linear measurements taken of the following: 1. Orbit length; 2. Orbit width; 3. Mandible height; 4. Mandibular fenestra height; 5. Mandibular fenestra length; 6. Post-orbital skull length; 7. Infratemporal fossa width; 8. Braincase length; 9. Retroarticular process length; 10. Rostrum length; 11. Dorsal skull length; 12. Interorbital width; 13. Palatal foramen width; 14. Infratemporal fossa width; 15. Supratemporal fossa maximum length; 16. Supratemporal fossa minimum width; 17. Supratemporal foramen length; 18. Supratemporal fenestra width; 19. Braincase width; 20. Skull width at quadrates; 21. Braincase height; 22. Foramen magnum width; 23. Pterygoid height; 24. Rostrum width at 2^nd^ maxillary alveolus; 25. Rostrum width at 4^th^ maxillary alveolus; 26. Rostrum width at anterior edge of palatine fenestra; 27. Palatal fossa length.

The volume or surface area of the paratympanic sinuses may be under the control of or constrained by other morphological variables (e.g., braincase volume, skull size, or tympanum surface area). Paratympanic sinus volume was regressed on other variables including a geometric mean for skull size (Fig 3), tympanum surface area, and braincase total volume. All variables were log transformed before performing a Reduced Major Axis regression in PAST [29]. The resulting regression estimates were then tested for a significant difference from isometry using the slope test function provided by SMATR for R [30,31]. By this test, the null of no significant difference from isometry is rejected if the test statistic returns a p-value < 0.05.

### Analysis of acoustic function

Treating the middle-ear volume as an acoustic resonator, we applied the Helmholtz equation for acoustic resonance (*f* = c/2π * (A_x_/L*V)^½^) to calculate a resonant frequency (*f*) for the contiguous middle-ear sinus system. We seek to test the hypothesis that the Helmholtz resonant frequency of the middle-ear space will correspond to biologically relevant frequencies. The air at a resonator opening behaves like a spring mass in simple harmonic motion, such that the frequency of resonance is equal to the vibration of the air in the container [32,33]. Using the Helmholtz equation given, we calculated this frequency using the speed of sound in air (c), the surface area of the resonator aperture (A), the volume of air in the resonator (V), and the effective length of the resonator opening (L). Two candidate apertures (A) for sound energy were tested. The apertures of the tympanic membrane and the subtympanic foramen were selected as reasonable vectors for air-borne sound energy, and the surface areas of these apertures (A_x_) were measured. For each candidate aperture tested, the effective resonator neck length (L) was calculated. For the subtympanic foramen, this was calculated as (*l* + γr), where (*l*) is the actual neck length and (r) is the radius of the unflanged aperture and the constant γ (= 1.4) represents the specific heat of compression for air in an adiabatic system. The calculation for the tympanic aperture was treated as a flanged aperture with an actual neck length of zero such that (L) was calculated as (γr) where γ = 1.7. This calculation was performed for all specimens in our ontogenetic series to see if the performance of the middle-ear as an acoustic resonator changed as a function of ontogeny.

## Results

### General pattern of pneumatic expansion

**Fig 4:**
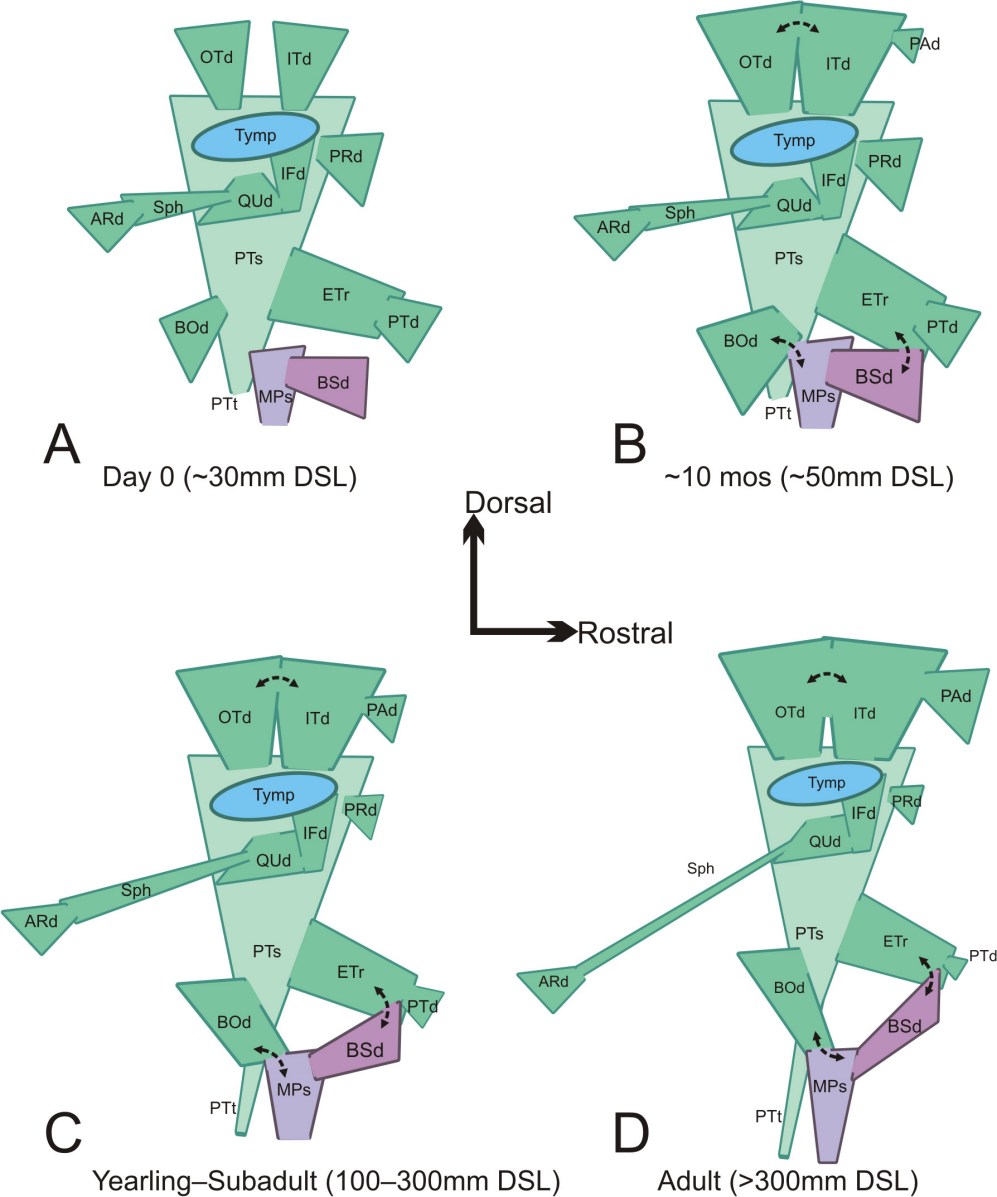
Schematic showing ontogenetic trends in the pharyngotympanic sinus system (green) and the median pharyngeal sinus system (purple) in right lateral view. Double ended arrows represent emergent confluences between diverticula and between the adjacent sinus systems. Ontogenetic stages are A. Day 0 (~30mm DSL), B. ~10 months (~50mm DSL), C. yearling to subadult (100–300mm DSL), and D. adult (>300mm DSL). **ARd**, articular diverticulum; **BOd**, basioccipital diverticulum; **BSd**, basisphenoid diverticulum; **ETr,** recessus epitubaricum; **IFd**, infundibular diverticulum; **ITd**, intertympanic diverticulum; **MPs**, median pharyngeal sinus; **OTd**, otoccipital diverticulum; **PAd**, parietal diverticulum; **PRd**, prootic diverticulum; **PTd**, pterygoid diverticulum; **PTs**, pharyngotympanic sinus; **PTt**, pharyngotympanic tube; **QUd**, quadrate diverticulum; **Sph**, siphonium; **Tymp**, tympanum.

Extant crocodylians have a dorsally directed evagination of the pharyngeal epithelium located immediately caudal to the choanae. Postnatally, this evagination is partially occluded by a plug of connective tissue which constricts its aperture. This connective tissue plug, referred to by Owen [2] as the “valvular membranous prominence,” fills the ventral part of the median pharyngeal tube (median Eustachian tube of Owen), but only slightly restricts the lateral passage of the pharyngotympanic tubes (see Table 2 for nomenclature). The valvular membranous prominence partly fills a small vestibule from which the pharyngotympanic tubes and the median pharyngeal tube ascend into the basicranium. These two passages (pharyngotympanic and median pharyngeal) reveal two discrete, but ultimately confluent pneumatic invasions into the braincase: the median pharyngeal system and the pharyngotympanic system (Fig 4). This duality of cranial pneumatization was noted by Simonetta [6], although he did not explicitly describe the details of the precise components and points of confluence of these two systems.

**Table 2:** Synonyms of paratympanic pneumatic structures used in this study and selected literature.

Study	Communicating tubes and parent sinuses			Diverticula		
This study	Median pharyngeal tube and sinus	Pharyngotympanic tube	Pharyngotympanic sinus	Intertympanic diverticulum	Basisphenoid diverticulum	Basioccipital diverticulum
Owen, 1850	Median Eustachian canal	Lateral Eustachian canal(s)	—	—	Basisphenoid branch of the median canal	Basioccipital branch of the median canal
Hasse, 1873	—	—	—	Antrum mastoideum	—	—
Chapman, 1894	Median Eustachian tube	Lateral Eustachian tubes proper		Supraoccipital passage	Anterior lateral passage	Posterior lateral passage
van Beneden, 1880	Canal intertympanique median	—	Cavum tympani	Cellules épitympaniques	Canal intertympanique antérieur	Canal intertympanique postérieur
Colbert, 1946	Median Eustachian tube	Lateral Eustachian tubes	Tympanic cavity	—	—	Rhomboid sinus
Simonetta, 1956	—	—	Vestibulum tubarum	—	Recessus intertympanicus	Vestibulum tubarum
Müller F, 1967	Mediane Eustach. Öffnung	Tuba Eustachii	Cavum tympani	Antrum mastoideum	Canalis anterior	Canalis posterior
Tarsitano, 1985	Median Eustachian Tube/ Hypophyseal-basicranial fenestra	Lateral Eustachian tubes	Tympanic cavity	Intertympanic sinus	Basisphenoid sinus	Basioccipital sinus

### Median pharyngeal system

Crocodylians have a median bony canal situated within the sutural contact between the basioccipital and the basisphenoid. This canal has been described as bifurcating to form rostral and caudal passages, each of which further bifurcate to form laterally directed passages [5,7,9]. Tarsitano [14] described this system of the median canal and its purported derivatives as being highly “verticalized” in adult alligators and as having the appearance of long and narrow tubes following a highly ventral-to-dorsal trajectory, although our results do not fully coincide with the characterization (see below).

**Fig 5:**
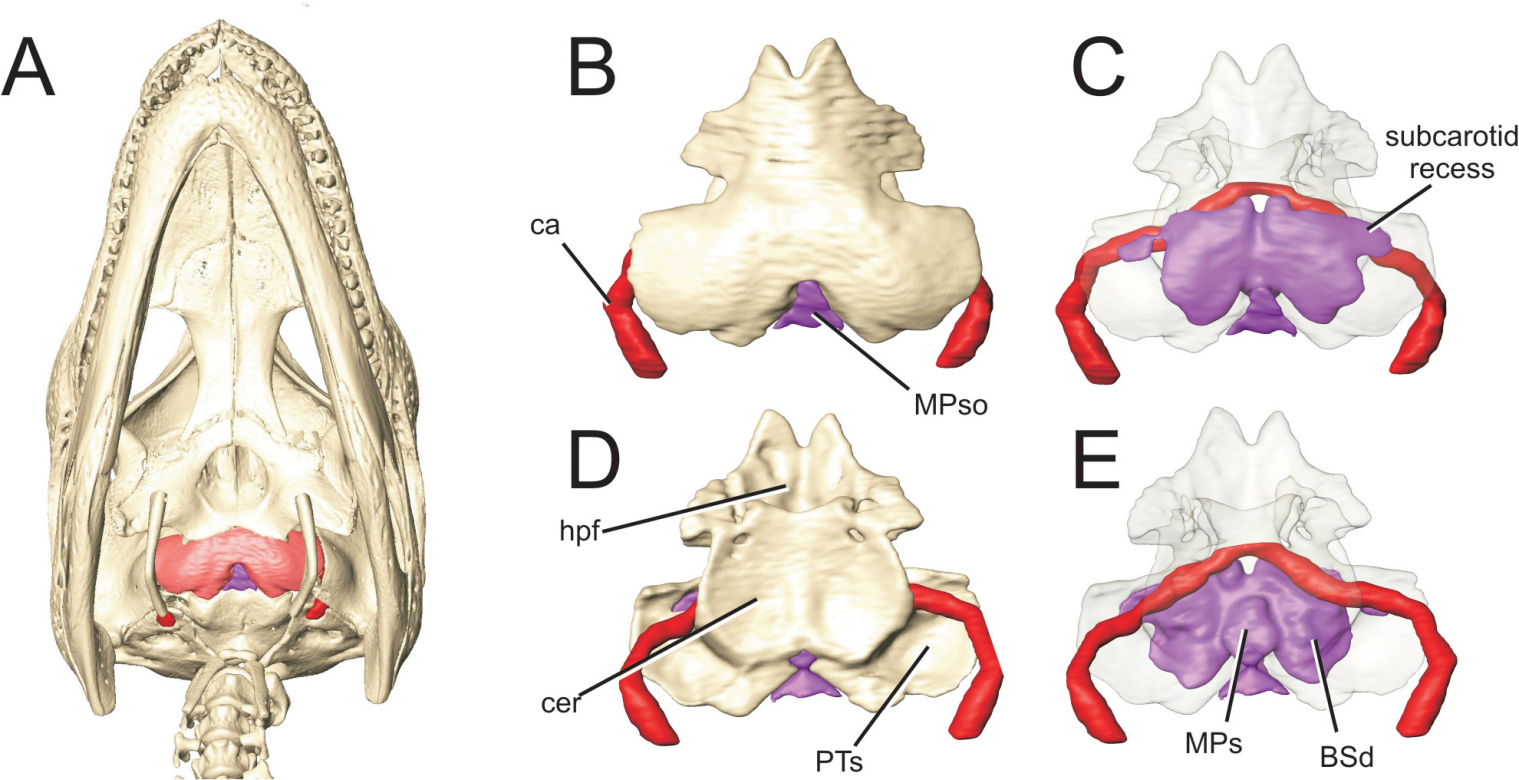
Perinatal *Alligator mississippiensis* (OUVC 10606) demonstrating the median pharyngeal sinus system. Rendered in A) ventral aspect with the basisphenoid in red. Isolated basisphenoid shown in B) ventral aspect and C) rendered transparent with the basisphenoidal diverticulum rendered in purple. Basisphenoid shown in D) dorsal aspect and E) rendered transparent. **BSd**, basisphenoid diverticulum; **ca**, carotid artery; **cer**, cerebral fossa; **hpf,** hypophyseal fossa; **MPs**, median pharyngeal sinus; **MPso**, ostium for median pharyngeal sinus; **PTs**, pharyngotympanic sinus.

The median canal itself is regarded as a remnant passage from the ascent of glandular oropharyngeal ectoderm to the hypophyseal fossa in early development (i.e., Rathke's pouch [10,14]; craniopharyngeal canal of birds [34, 35]). Subsequently, this remnant epithelial pocket undergoes pneumatic expansion, but only accounts for some of the passages previously ascribed to it. The pneumatic expansion of the median pharyngeal canal yields two rostrolateral inflations, each inflating its side of the basisphenoid. Early in postnatal ontogeny (perinatal day 0–ca. 25 mm DSL) these paired basisphenoid diverticula are horizontally expanded and coplanar with the laminae of the basisphenoid, having a dorsoventrally oblate conformation (Figs 4 and 5).

At this early stage, there are no communications between the basisphenoid diverticula and any other diverticula or sinus system. This situation changes rapidly later in ontogeny, and by the time an alligator has reached about 33 mm DSL, the basisphenoid diverticula have achieved a number of communications with the adjacent pharyngotympanic sinus system. These include a caudodorsally extended subcarotid recess (Fig 5C), which communicates with the pharyngotympanic sinus at the proximal terminus of the pharyngotympanic tube, and a rostrolateral communication with the junction of the recessus epitubaricum of the pharyngotympanic sinus [34] and its derivative, the pterygoid diverticulum. Late in ontogeny, particularly in individuals approaching the asymptote of growth, the subcarotid recess becomes increasingly elongate and tubular, ultimately constricting distally to the extent that it loses its communication with the pharyngotympanic sinus. The basisphenoid diverticula themselves become slightly less dorsoventrally flattened later in ontogeny, but generally conform to the shape of the basisphenoid. The paired basisphenoid diverticula ultimately converge to a shared median communication with the median pharyngeal canal, and this passage (itself a remnant of Rathke's pouch[34]; basisphenoid branch of Owen [2]; anterior communicating canal of Chapman [9]) is also only very slightly vertically elongated late in ontogeny. Within the median pharyngeal sinus system, only the median pharyngeal canal exhibits any large degree of verticalization, becoming approximately four times taller than wide. The basisphenoid diverticula have been figured and described as being tubular passages (e.g., lateral anterior communicating branches of Chapman [9]) but, in fact, at no ontogenetic stage do they have a tubular form, even under the influence of verticalization. This phenomenon is analyzed further in the Discussion.

### Pharyngotympanic system

**Fig 6:**
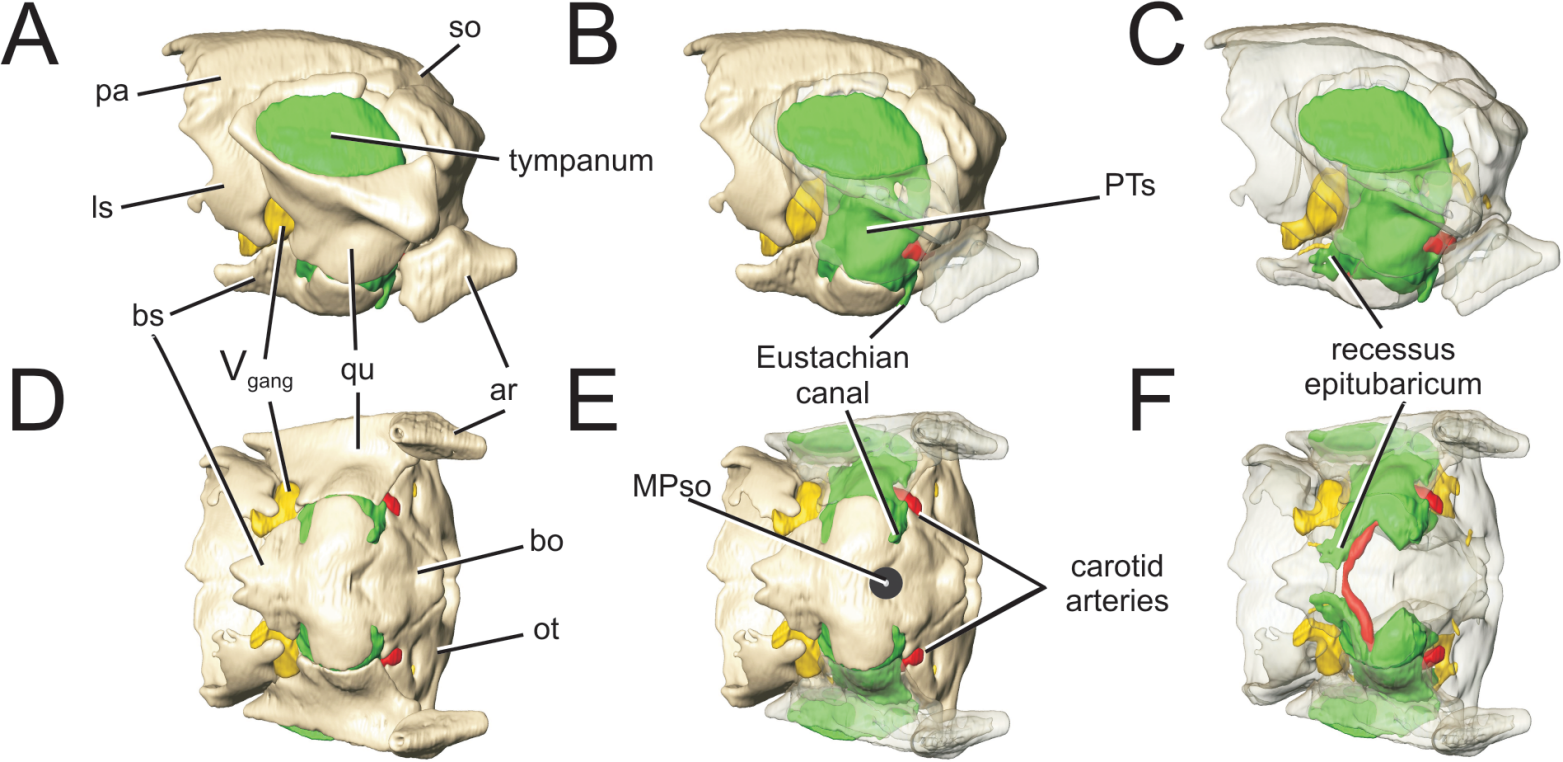
Perinatal *Alligator mississippiensis* (OUVC 10606) demonstrating the pharyngotympanic sinus. Isolated braincase shown in A) left lateral view with B) the suspensorium rendered transparent and C) all bones rendered transparent. Also in D) ventral aspect with E) suspensorium rendered transparent and F) all bones rendered transparent. Pharyngotympanic sinus is rendered in green. **ar**, articular; **bo,** basioccipital; **bs**, basisphenoid; **ls**, laterosphenoid; **MPso**, ostium for median pharyngeal sinus; **pa**, parietal; **PTs**, pharyngotympanic sinus; **qu,** quadrate; **V**_**gang**_, trigeminal ganglion.

This is the principal pneumatic system of the sauropsid middle ear, being comprised in crocodylians by the pharyngotympanic sinus and its diverticula (Fig 4). Like the median pharyngeal system, it arises as an evagination of the pharyngeal epithelium. The pharyngotympanic system is represented by paired inflations whereby each middle-ear space communicates with the pharynx via the pharyngotympanic tube. Each canal comprises a passage through the basioccipital-basisphenoid suture, leading to foramina situated caudolateral to those of the median pharyngeal canal (Fig 6). The pharyngotympanic sinus is bounded medially by the prootic and otoccipital, and is enclosed ventrolaterally by the quadrate. Ventrally, the pharyngotympanic sinus occupies a shallow fossa on the basisphenoid, and dorsolaterally it is covered by the tympanum. The pharyngotympanic sinus gives rise to seven discrete diverticula and also the recessus epitubaricum, all of which are present in neonate specimens (Fig 4). Two of these secondary diverticula themselves give rise to accessory diverticula. During the course of ontogeny, several of these diverticula and accessory diverticula develop communications between one another and with portions of the adjacent median pharyngeal sinus system.

### Basioccipital diverticulum

**Fig 7:**
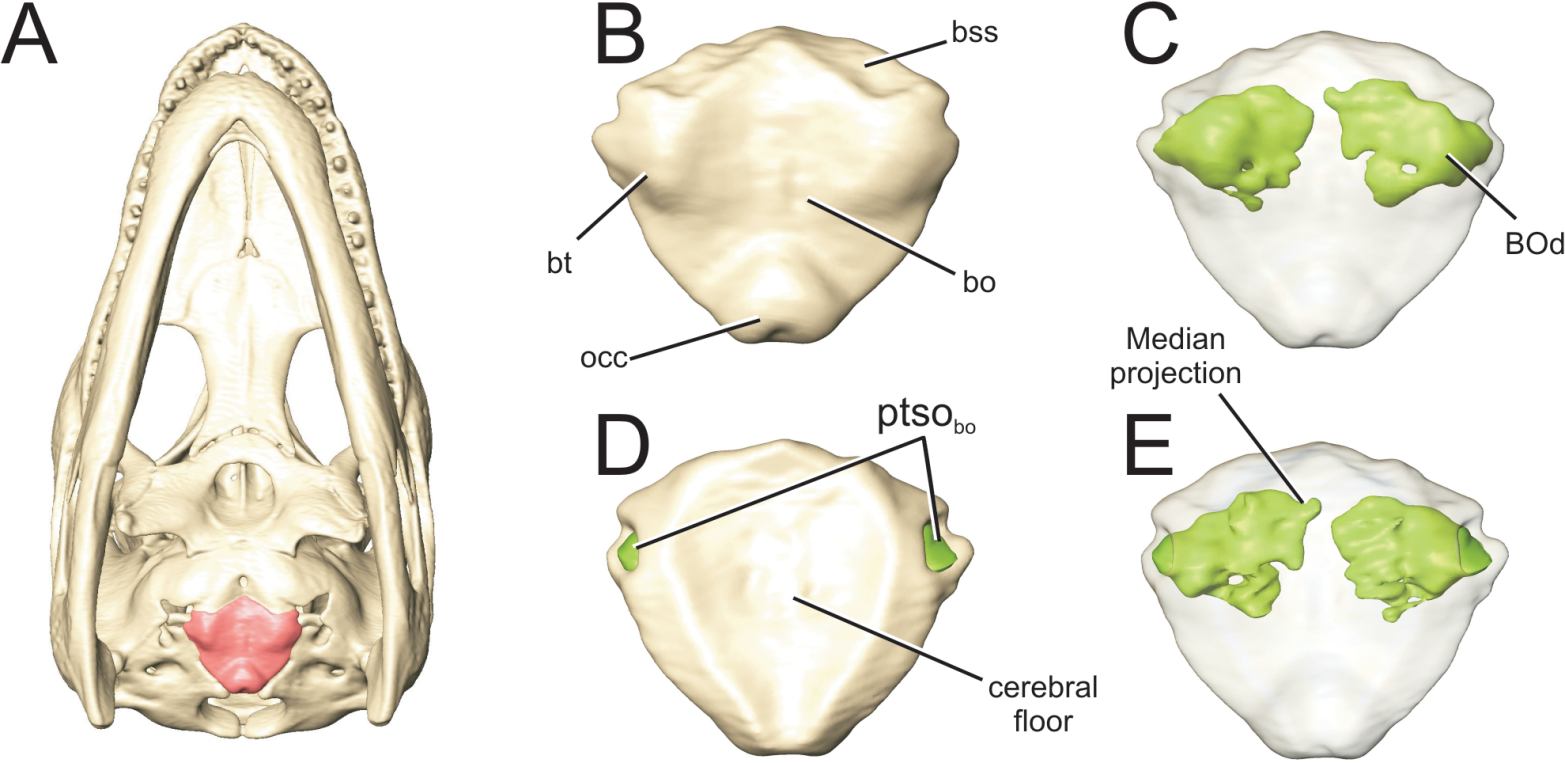
Perinatal *Alligator mississippiensis* (OUVC 10606) shown in A) ventral aspect with the basioccipital in red. Isolated basioocipital shown in B) ventral aspect and C) rendered transparent with the basisoccipital diverticulum rendered in green. And in D) dorsal aspect and E) rendered transparent with the basioccipital diverticulum rendered in green. **bo**, basioccipital; **BOd**, basioccipital diverticulum; **bss,** basisphenoidal suture; **bt**, basal tuber; **occ,** occipital condyle; **ptso**_bo_, communicating ostium between pharyngotympanic sinus and basioccipital diverticulum.

Extending from the pharyngotympanic sinus just dorsomedial to the pharyngotympanic tube and ventral to the internal carotid artery (Fig 4), the basioccipital diverticulum invades the basioccipital bone via a foramen in its rostrolateral apex and excavates the precondylar portion of that skeletal element (Fig 7). This diverticulum is ventromedially and slightly caudally projected from its point of origin and does not communicate with any adjacent diverticula or sinus systems in neonates. Postnatally, this diverticulum converges rostromedially and slightly ventrally with its contralateral counterpart, at which point the conjoined diverticula immediately intersect the median pharyngeal recess at the basioccipital-basisphenoid suture. The sequential ontogenetic appearance of this diverticulum relative to the basisphenoidal diverticulum of the median pharyngeal sinus system has been noted previously by Müller [10], but in that study the basioccipital diverticulum was grouped with the median pharyngeal system (Intertympanales System of Müller) implying a common derivation. Even in very large adult individuals, the convergence itself (termed the posterior communicating branch by Chapman [9]) is not verticalized.

### Recessus epitubaricus

This recess is a rostral extension of the pharyngotympanic sinus (Fig 4) that in crocodylians is made distinct from its parent sinus by a constriction formed by the ventrally-bounding internal carotid artery and the dorsally-bounding trigeminal ganglion (Fig 6E). This recess was noted in neonate alligators by Simonetta [6], who also suggested its homology with that part of the sauropsid middle ear bounded dorsally by the *mm*. *levator* and *protractor pterygoideus* and the basipterygoid process rostroventrally. Recent investigations of the middle-ear space in sauropsids [36] reinforce this hypothesis of homology. In crocodylians, however, incorporation of the suspensorium into the lateral braincase wall has enclosed the recessus epitubaricus and eliminated its association with the cavum epiptericum and the protractor muscles, which have been lost [37,38]. The recessus epitubaricus is bounded laterally by the quadrate and medially by the prootic. Rostromedially the recess excavates the basisphenoid until it is constrained medially by the hypophyseal fossa. This recess communicates with two adjacent diverticula. In perinatal specimens, there is a rostrolaterally directed diverticulum derived from the mid-point of the recessus epitubaricus which invades the pterygoid. Additionally, the recessus epitubaricus has a ventrolateral communication with the basisphenoid diverticulum of the median pharyngeal sinus system, but this communication is not present in perinatal individuals.

### Pterygoid diverticulum

**Fig 8:**
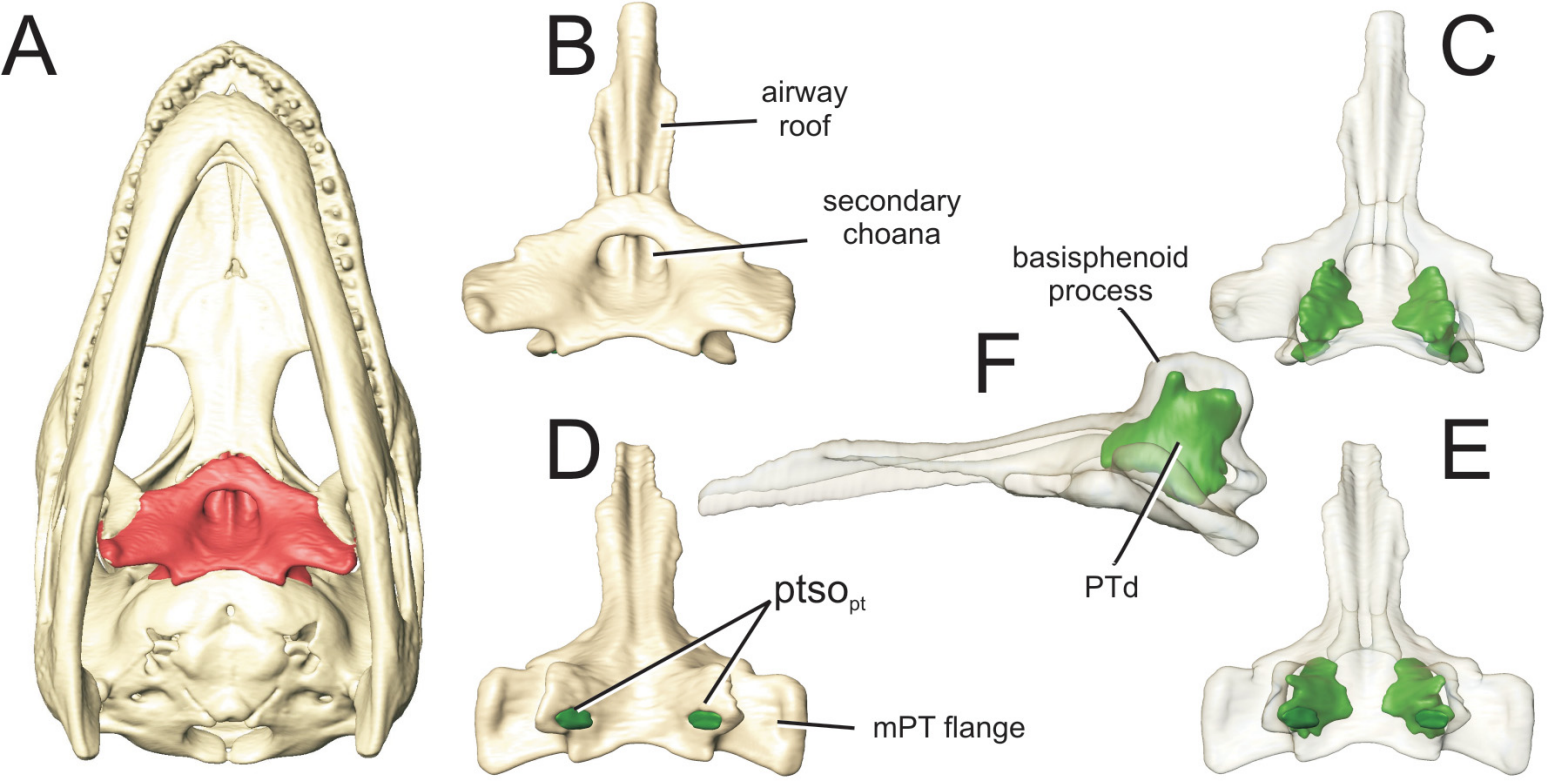
Perinatal *Alligator mississippiensis* (OUVC 10606) shown in A) ventral aspect with the pterygoid in red. Isolated pterygoid shown in B) ventral aspect and C) rendered transparent with the pterygoid diverticulum (PTd) rendered in green. Pterygoid shown in D) dorsal aspect and E) rendered transparent. F) Left lateral transparent rendering of pterygoid. **mPT,** pterygoideus muscle; **PTd**, pterygoid diverticulum; **ptso**_**pt**_, communicating ostium between pharyngotympanic sinus and pterygoid diverticulum.

As mentioned above, this diverticulum is derived from the recessus epitubaricum (Fig 4), with which it maintains a communicating ostium crossing the pterygoid-basisphenoid suture (Fig 8). This diverticulum is figured in Simonetta [6], but is not recognized as a separate extension of the recessus epitubaricum. However, there is a clear constriction between this diverticulum and its parent, and a foramen penetrating the medial aspect of the basisphenoid process of the pterygoid at its sutural contact with the basisphenoid is the osteological correlate indicating the infiltration of the pterygoid by this diverticulum. The pterygoid diverticulum conforms to the general shape of the pterygoid such that it bears dorsal, rostrolateral, and lateral projections corresponding to the basisphenoid process, choanal margin, and lateral process of the pterygoid, respectively. During ontogeny, the pterygoid diverticulum is reduced, with the rostrolateral projection being the first to be completely obliterated. Upon reaching an adult size, the remaining portions of the pterygoid diverticulum are also greatly reduced (Fig 2D), to the point that in most large individuals (> ~300 mm DSL) they are entirely absent.

### Prootic diverticulum

**Fig 9:**
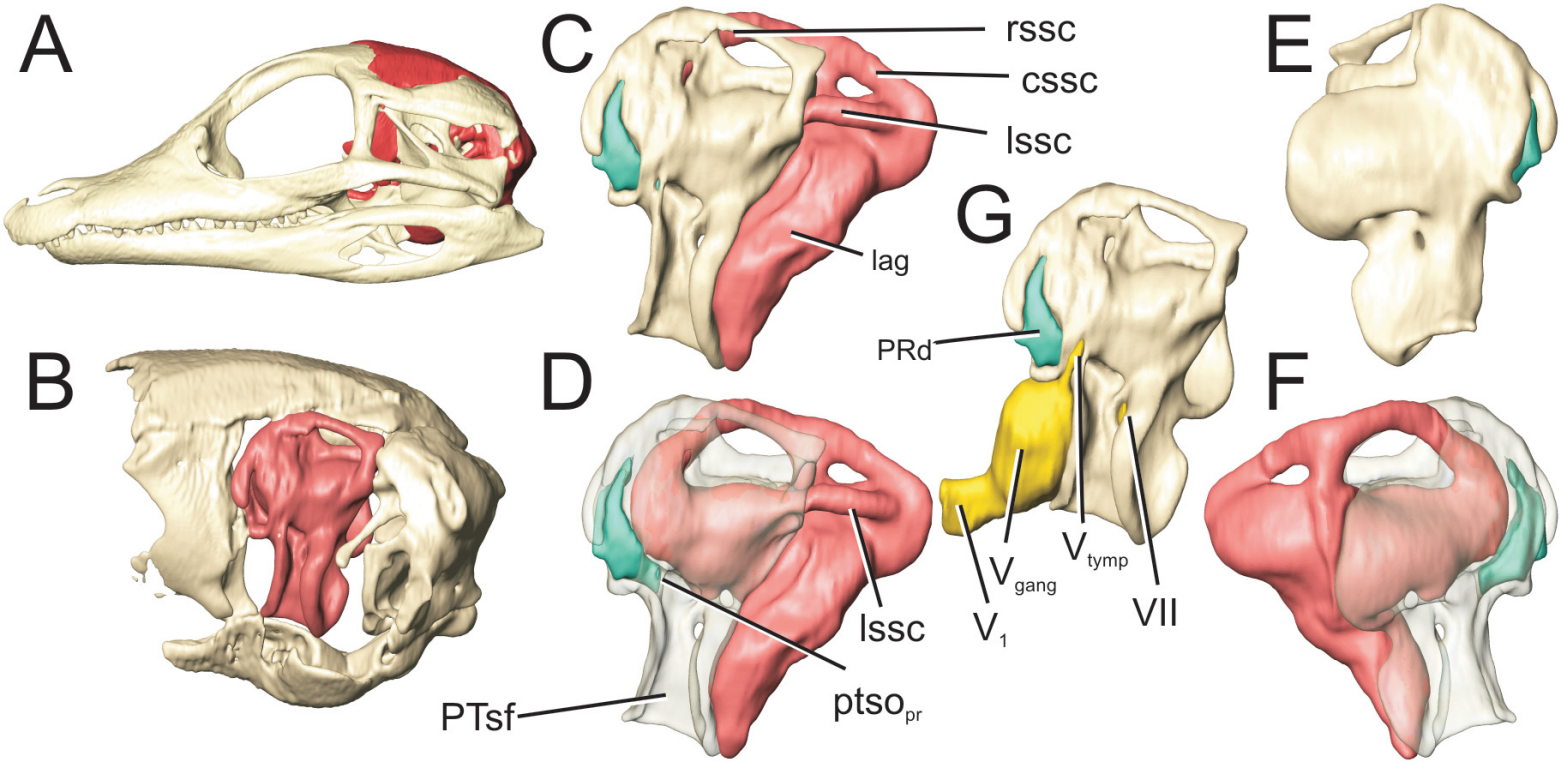
Perinatal *Alligator mississippiensis* (OUVC 10606) shown in A) left lateral view with the braincase rendered in red and B) with the braincase isolated and the prootic rendered in red. Left lateral view of C) the isolated prootic, D) rendered transparent with endosseous labyrinth and G) with associated cranial nerves. Left prootic in E) medial aspect and F) rendered transparent with endosseous labyrinth. Labyrinth is rendered in pink, and the prootic diverticulum rendered in teal. **cssc**, caudal semicircular canal; **lag**, lagena; **lssc**, lateral semicircular canal; **PRd**, prootic diverticulum; **PTsf**, fossa for pharyngotympanic sinus; **ptso**_**pr**_, communicating ostium between pharyngotympanic sinus and prootic diverticulum; **rssc**, rostral semicircular canal; **V**_**gang**_, trigeminal ganglion; **V**_**tymp**_, tympanic ramus of trigeminal nerve; **V**_**1**_, ophthalmic ramus of trigeminal nerve; **VII**, facial nerve.

This diverticulum creates a foramen in the prootic rostral and just dorsolateral to the foramen for cranial nerve VII, and just ventromedial to the foramen for the tympanic branch of cranial nerve V (Fig 9). It excavates the rostral part of the prootic and is constrained laterally by the tympanic branch of CN V, ventrally by the ganglion of CN V and caudally by the ampulla of the rostral semicircular canal. In perinatal individuals the prootic diverticulum has no communications with any other diverticulum, and is confined to the rostral portion of the prootic (Fig 4A). Later in ontogeny two projections of this diverticulum exit the prootic. In individuals larger than ~80 mm DSL, a rostral extension of the prootic diverticulum passes into the laterosphenoid via a small foramina at the ventral edge of the abutting contact between the prootic and laterosphenoid which exists just dorsal to the trigeminal ganglion. Late in ontogeny, the ostium to the laterosphenoid expansion becomes constricted, isolating this projection of the diverticulum and is completely obliterated in very large individuals (Fig 4D). A dorsal projection of the prootic diverticulum exits the prootic just medial to the sulcus for the tympanic branch of CN V and rostromedial to the ampulla for the rostral semicircular canal (Fig 9). The resulting foramen opens into the lateral wall of a recess excavated by the pharyngotympanic sinus into the prootic rostral to the rostral semicircular canal, resulting in a re-entrant pathway to the pharyngotympanic sinus.

### Pneumatic inflations of the suspensorium

**Fig 10:**
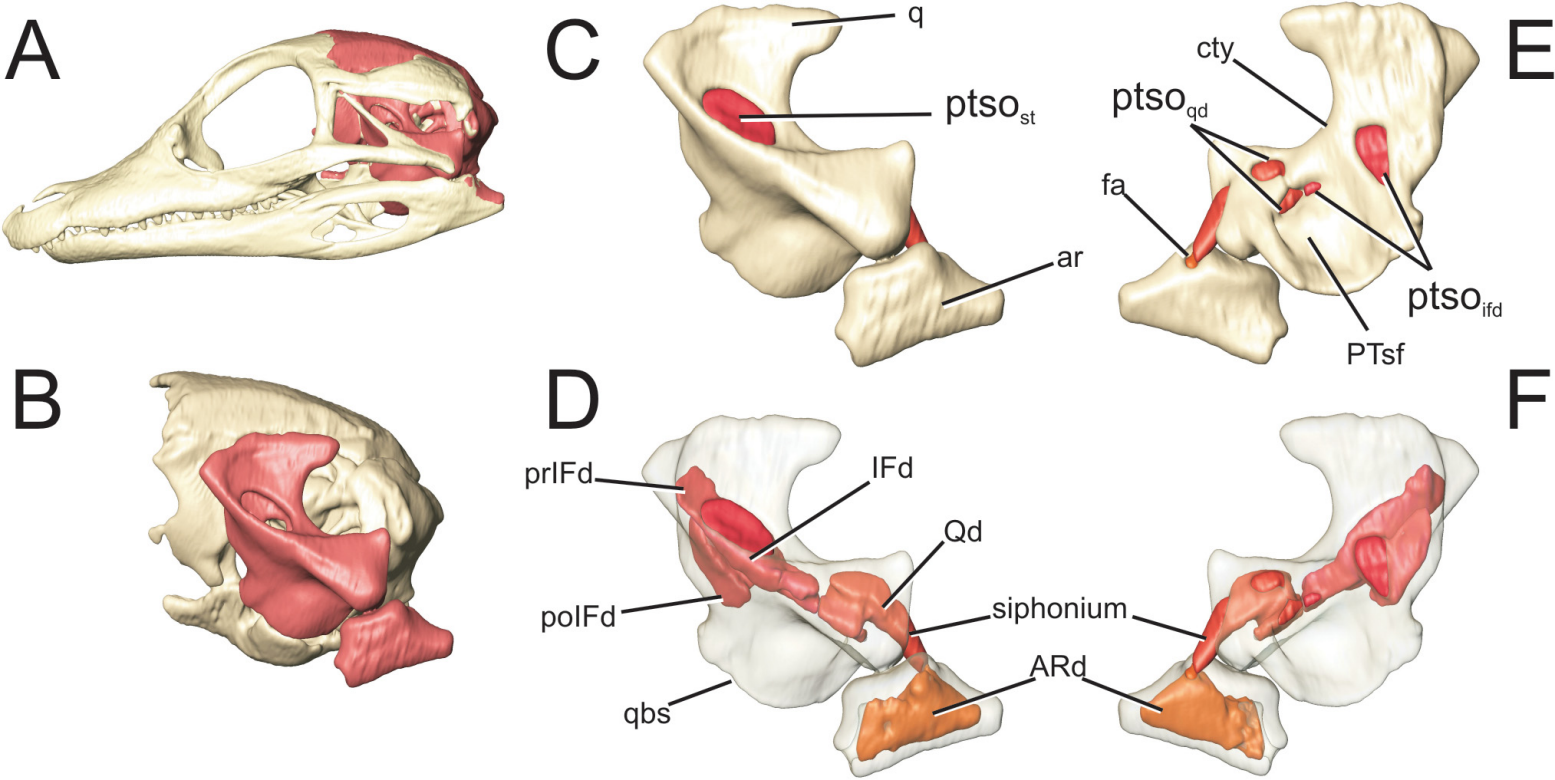
Perinatal *Alligator mississippiensis* (OUVC 10606) shown in A) left lateral view with the braincase rendered in red and B) with the braincase isolated and the suspensorium rendered in red. Isolated suspensorium in C) left lateral view and D) rendered transparent. Suspensorium in E) medial aspect and F) rendered transparent. **ar**, articular; **ARd**, articular diverticulum; **cty**, crista tympanicum; **fa**, foramen aerum; **IFd**, infundibular diverticulum; **poIFd**, postinfundibular recess; **prIFd**, preinfundibular recess; **ptso**_ifd_, communicating ostium between pharyngotympanic sinus and infundibular diverticulum; **ptso**_**qd**_, communicating ostium between pharyngotympanic sinus and quadrate diverticulum; **ptso**_**st**_, communicating ostium infundibular diverticulum and the subtympanic portion of the pharyngotympanic sinus; **qbs**, = basisphenoidal suture of quadrate; **q**, quadrate; **Qd**, quadrate diverticulum.

Two separate but confluent diverticula invade the quadrate (Fig 4): the infundibular and quadrate diverticula are diagnosed by a series of foramina that are situated just ventromedial to the crista tympanica (Fig 10) and perforate the medial surface of the quadrate. The rostralmost of these foramina sits at the apex of a funnel-shaped recess (infundibulum) that widens dorsally and ultimately opens to an aperture just deep to the tympanum (subtympanic foramen). The association of this conical infundibulum with the tympanum has the potential to inform us about particular acoustic parameters, which will be addressed in greater detail in the Discussion. The infundibular diverticulum expands into two recesses: the rostrally situated preinfundibular recess which inflates the rostromedial corner of the quadrate, and the caudolaterally situated postinfundibular recess. The postinfundibular recess communicates caudally with the quadrate diverticulum, and also with the pharyngotympanic sinus medially. On the medial surface of the quadrate, just caudal to the infundibular foramen, the pharyngotympanic sinus excavates a wide but shallow fossa. The dorsal apex of this fossa leads to two foramina, a rostral foramen leading to the infundibular diverticulum and a more caudal foramen to the quadrate diverticulum. Late in ontogeny, the quadrate diverticulum and the postinfundibular recess of the infundibular diverticulum become increasingly confluent. Dorsal to the paired openings to the postinfundibular recess and quadrate diverticulum, and just ventral to the crista tympanicum, is a larger foramen leading to the main inflation of the quadrate diverticulum. This diverticulum exits the quadrate just caudal to the pedicle contacting the otoccipital and squamosal (Fig 10E). Throughout ontogeny, the resulting foramen aerum opens in the same position relative to the pedicle. The pedicle itself and the condylar portion of the quadrate become more massive relative to the portion of the quadrate bearing the tympanum and fossa for the middle ear. Consequently, the part of the quadrate diverticulum that passes deep to the pedicle is elongated into a cylindrical tube or siphonium. This siphonial elongation first appears in individuals of ~50 mm DSL (Fig 4B) and is about 10% of the combined length of the infundibular and quadrate diverticula. This proportion increases throughout ontogeny to the extent that in very mature individuals the part of the siphonium contained by the quadrate reaches up to 300% of the combined length of the infundibular and quadrate diverticula (Fig 4D). The epithelial siphonium leaves the quadrate and passes deep to *m*. *depressor mandibuli*, ultimately producing a foramen in the dorsomedial edge of the articular bone just caudal to the glenoid fossa (Fig 10E). Early in ontogeny, the resulting articular diverticulum inflates the entire articular bone, conforming to the shape of that element. In perinatal individuals, this diverticulum occupies slightly above 50% of the total bone volume. During the course of ontogeny, however, the diverticulum occupies a proportionately smaller percentage of the bone volume. This proportion can be as small as 2% of the total volume in large individuals, and in senescent individuals the communication with the siphonium can be very restricted or entirely occluded, resulting in obliteration of this diverticulum.

### Otoccipital diverticulum

**Fig 11:**
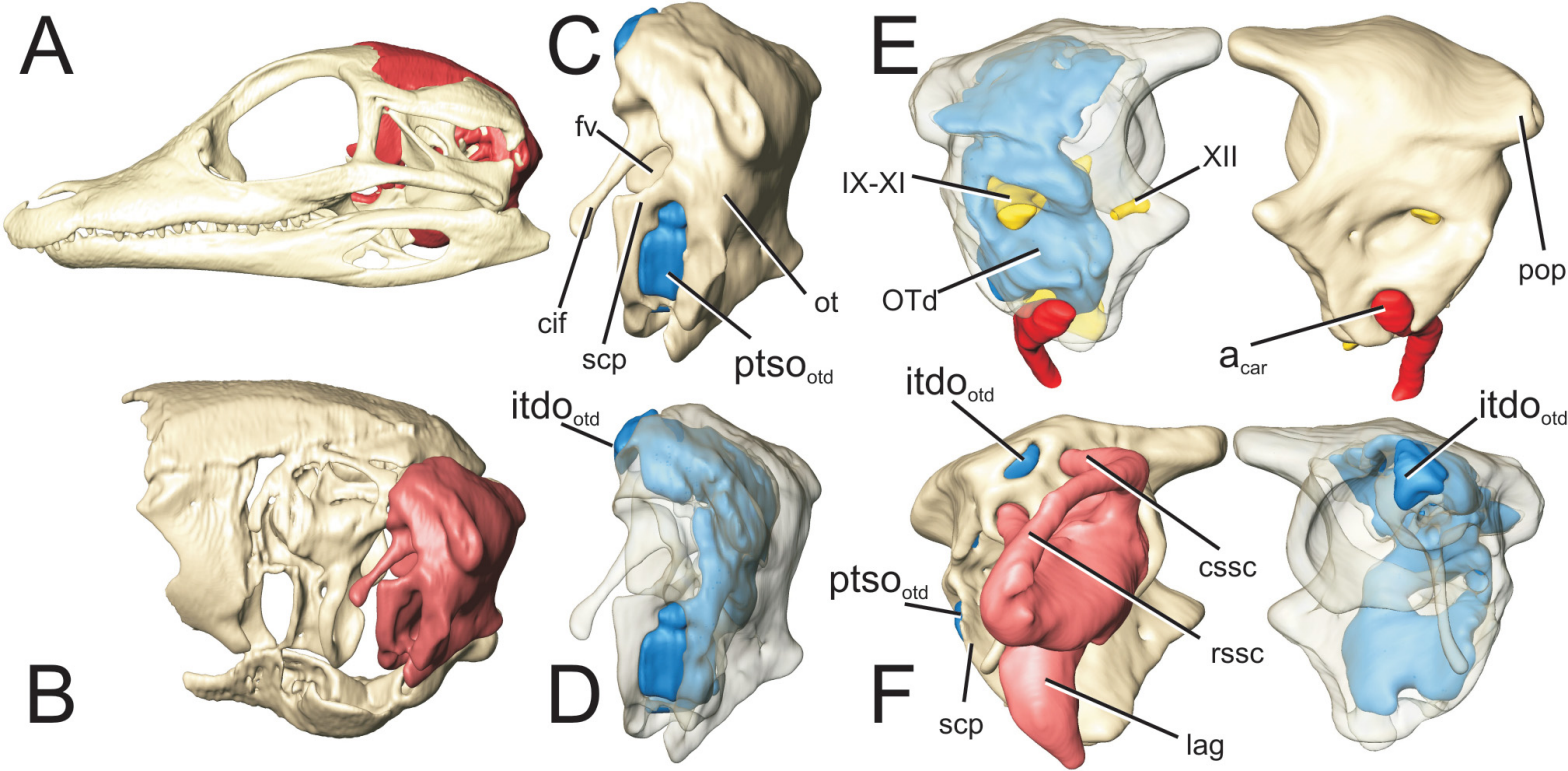
Perinatal *Alligator mississippiensis* (OUVC 10606) shown in A) left lateral view with the braincase rendered in red and B) with the braincase isolated and the otoccipital rendered in red. Isolated otoccipital C) in left lateral view and D) rendered transparent. Both otoccipitals in E) caudal aspect with the left otoccipital rendered transparent and F) in anterior aspect with the left otoccipital rendered transparent. Labyrinth rendered in pink, otoccipital diverticulum rendered in blue. **a**_**car**_, carotid artery; **cif**, crista interfenestralis; **cssc,**caudal semicircular canal; **fv**, fenestra vestibularis; **itdo**_**otd**_, communicating ostium between intertympanic diverticulum and otoccipital diverticulum; **IX-XI,** combined pathway for glossopharyngeal, vagus, and spinal accessory nerves; **lag**, lagena; **ot**, otoccipital; **OTd**, otoccipital diverticulum; **pop**, paroccipital process; **ptso**_**otd**_, communicating ostium between pharyngotympanic sinus and otoccipital diverticulum; **rssc**, rostral semicircular canal; **scp**, subcapsular process; **XII,** hypoglossal nerve.

At the ventrolateral edge of the otoccipital bone, between the subcapsular process and the suture for the quadrate is an aperture that is intersected by the carotid artery. This aperture provides the communication between the pharyngotympanic sinus and the otoccipital diverticulum (Figs 4 and 11). Medial to this aperture, the otoccipital diverticulum inflates the ventral part of the otoccipital bone, its dorsal progress being interrupted by the common passage conducting the jugular vein and CN IX-XI. This passage is only partially ossified in perinatal individuals but is completely ossified in individuals larger than ~50 mm DSL. The otoccipital diverticulum, as it expands dorsally, surrounds this neurovascular bundle, passing around it both medially and laterally. Dorsal to the neurovascular passage, the diverticulum conforms to the boundaries of the paroccipital process of the otoccipital, and exits this bony element via a foramen between the openings for the horizontal and caudal semicircular canals. In individuals larger than ~50 mm DSL, the otoccipital diverticulum intersects the intertympanic diverticulum, the communication becoming broadly confluent later in ontogeny (Fig 4) such that it is hard to appreciate in adult specimens that these diverticula were previously separate. Additionally, in perinatal individuals a second communication with the intertympanic diverticulum develops medial to the passage of the caudal semicircular canal. In adults, the portion of the otoccipital diverticulum ventral to the neurovascular canal and dorsal to the carotid canal is obliterated.

### Intertympanic diverticulum

**Fig 12:**
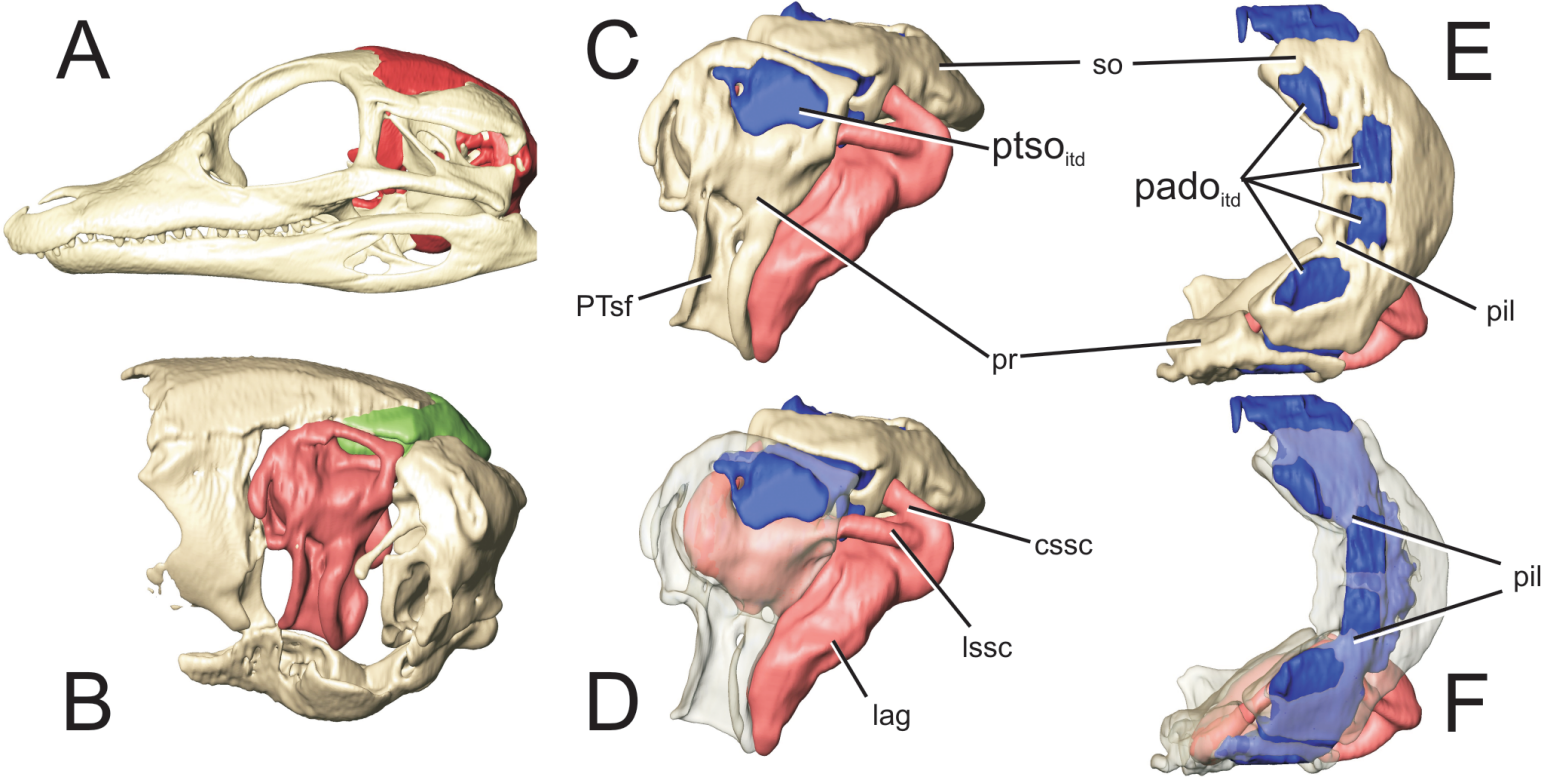
Perinatal *Alligator mississippiensis* (OUVC 10606) shown in A) left lateral view with the braincase rendered in red and B) with the braincase isolated and the prootic rendered in red and the supraoccipital rendered in green. Isolated prootic and supraoccipital with endosseous labyrinth C) in left lateral view and D) with the left prootic rendered transparent. Isolated prootic and supraoccipital E) in dorsal view and F) with the supraoccipital and left prootic rendered transparent. Labyrinth rendered in pink, intertympanic diverticulum rendered in blue. **cssc,**caudal semicircular canal; **lag**, lagena; **lssc**, lateral semicircular canal; **pado**_**otd**_, communicating ostia between parietal diverticula and intertympanic diverticulum; **pil**, osseous pillar; **pr**, prootic; **PTsf**, fossa for pharyngotympanic sinus; **ptso**_**itd**_, communicating ostium between pharyngotympanic sinus and intertympanic diverticulum; **so**, supraoccipital.

This diverticulum enters the braincase by perforating the prootic dorsal to the ampulla of the horizontal semicircular canal and caudal to the ampulla of the rostral semicircular canal (Figs 4 and 12). This opening and the recess excavated by this diverticulum were first compared to the mammalian mastoid air cells by Cuvier [1], resulting in the label “mastoid antrum” applied by Hasse [3]. This term enjoys particular currency in the recent literature, especially in phylogenetic analyses citing Clark [16]. We use the term 'intertympanic' diverticulum, proposed by Tarsitano et al. [39], which more accurately describes the nature of this diverticulum, rather than promulgate the use of the term “mastoid antrum” in crocodylomorphs and run the risk of confusion with the non-homologous mammalian mastoid antrum.

The ostium for this diverticulum is just lateral to the suture between the prootic and the supraoccipital. Medial to this suture, the intertympanic diverticulum passes into the supraoccipital. The diverticular inflation of the supraoccipital begins with the lateral aspect and proceeds medially until the expanding diverticulum is constrained by the semicircular canals. On reaching the rostral semicircular canal, the diverticulum is projected slightly rostrally and dorsomedially over the canal, exiting the supraoccipital. Similarly, the caudal semicircular canal and the crus communis constrain the caudomedial expansion of the diverticulum such that there is a constriction leading into the medial portion of the supraoccipital. Here, the diverticular inflation meets the inflation from the contralateral side. This intertympanic communication is incipient in perinatal individuals, but persistent for the remainder of ontogeny. Each half of this medial inflation exits the supraoccipital rostrodorsally. At the caudal aspect of the prootic-supraoccipital junction, just lateral to the caudal semicircular canal, the intertympanic diverticulum communicates with the otoccipital diverticulum. This communication again is incipient in perinatal individuals, and becomes increasingly broad and confluent later in ontogeny (Fig 4). Additionally, a second communication between the otoccipital and intertympanic diverticula develops medial to the caudal semicircular canal in individuals larger than ~50 mm DSL, becoming increasingly confluent with age.

### Parietal diverticulum

**Fig 13:**
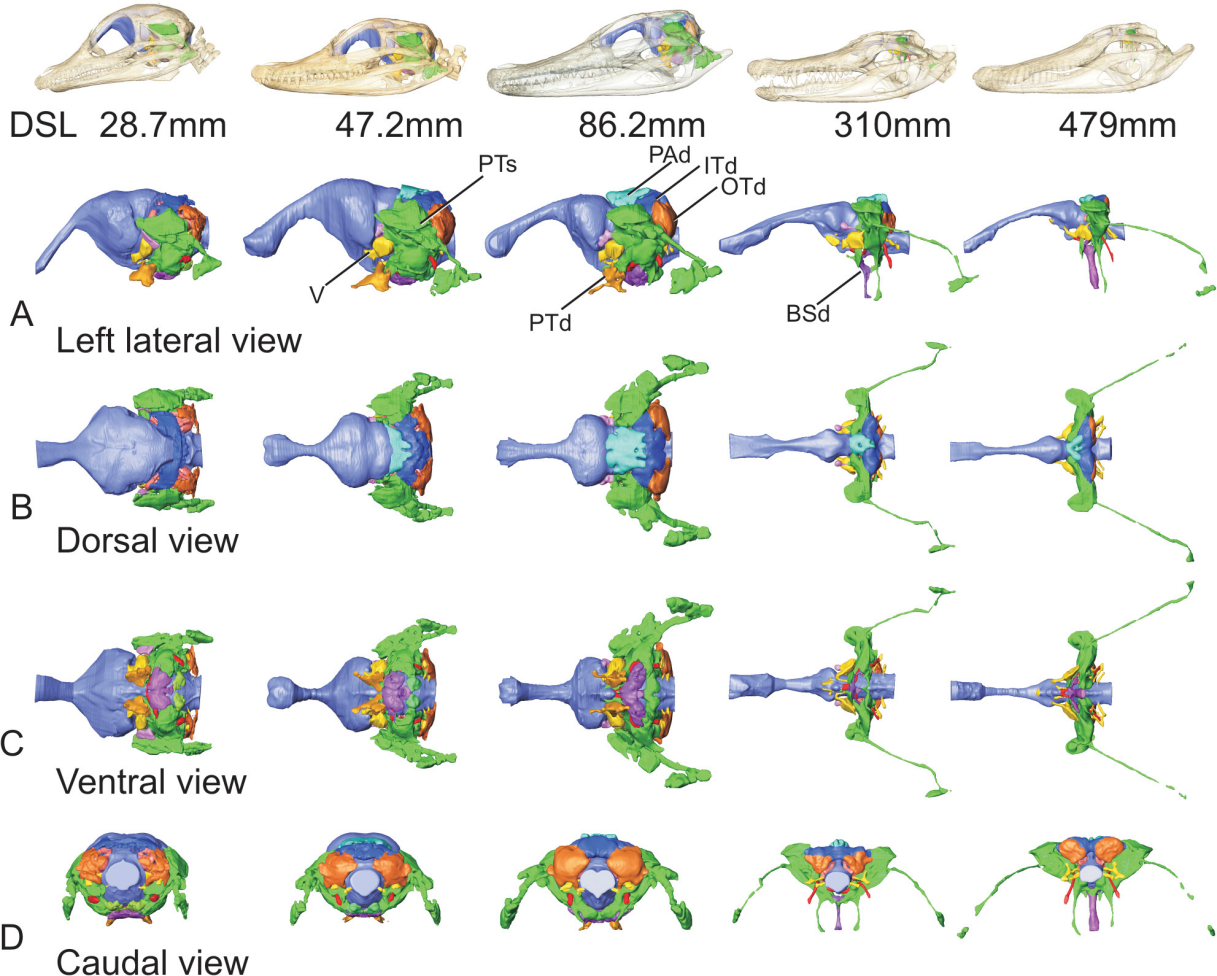
Ontogenetic series of *Alligator mississippiensis* showing the skulls rendered transparent, the brain endocast in blue, and the middle ear sinuses and component diverticula in various other colors. Columns pertain to different views of the same specimen. Specimens illustrated at unit scale to emphasize changing relative sizes of components. Rows: A) left lateral view; B) dorsal view; C) ventral view; D) caudal view. **BSd**, basisphenoid diverticulum; **ITd**, intertympanic diverticulum; **OTd**, otoccipital diverticulum; **PAd**, parietal diverticulum; **PTd**, pterygoid diverticulum; **V**, trigeminal nerve.

In individuals larger than ~50 mm DSL (Fig 4B), those parts of the intertympanic diverticulum exiting the supraoccipital excavate the parietal (Fig 13). The medial and lateral excavations conjoin with their contralateral counterparts and very rapidly become confluent with one another. The result of these infiltrations and confluences is the appearance of three pairs of foramina on the parietal that are mirrored about the midline. The two more medial pairs of foramina occur at the supraoccipital-parietal suture, and the lateralmost pair of foramina is situated at the prootic-parietal junction. The rostrolateral projections of the parietal diverticulum (corresponding to the lateralmost pair of foramina) are constrained laterally by the rostral semicircular canals, circumscribing this structure rostrally where it intersects the pharyngotympanic sinus adjacent to the re-entrant junction of the prootic diverticulum.

### Allometric relationships

**Fig 14:**
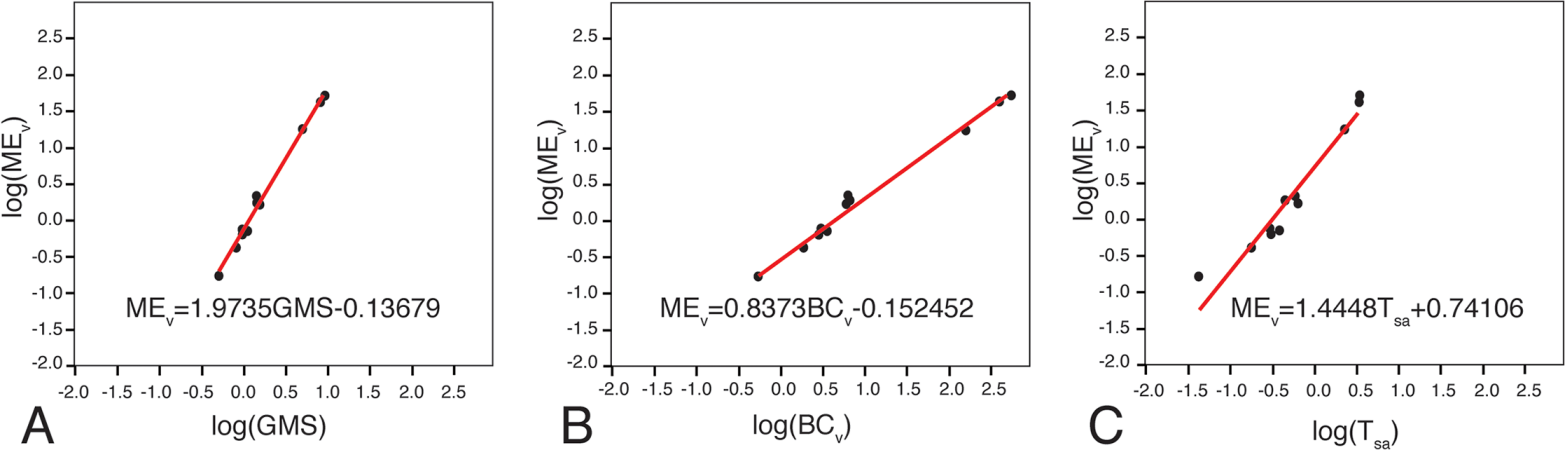
Comparisons of reduced major axis (RMA) regressions for middle-ear volume regressed onto A) skull size, B) braincase volume, C) tympanum surface area. BC_v_ = braincase volume; B_v_ = brain volume; GMS = geometric mean of skull-size measurements; M_ev_ = middle-ear volume; T_sa_ = tympanum surface area.

We performed regressions to discover any particular constraint or correlation that accounts for sinus morphology. In order to distinguish isometric relationships, we would expect a slope of 3 for regressions of middle-ear volume on a linear measurement, (*e*.*g*. GMS of skull size), a slope of 1.5 when regressing middle-ear volume on tympanic surface area, and a slope of 1 when regressing middle-ear volume on another volumetric measure such as braincase volume. We determined that the volume of the middle-ear sinuses is highly correlated with skull size, braincase volume, and tympanum surface area (Table 3, Fig 14). Middle-ear sinus volume is in negative allometry with respect to both braincase volume and skull size. We measured braincase volume to include parts of the suspensorium. Here also the relative size of the insertion points for the increasing jaw adductor muscle mass seems to be reflected in the allometric relationship between the postorbital head (braincase) volume relative to the middle ear contained within the braincase. The surface area of the tympanum, however, is in isometry with middle-ear volume.

**Table 3:** Comparisions of reduced major axis (RMA) regressions for middle-ear volume.

Regression	r^2^	Slope	95% confidence on slope	c	intercept
ME_v_ on GMS	0.9925	1.9735*	1.8157–2.0548	0.99626	-0.13679
ME_v_ on BC_v_	0.9894	0.8273*	0.7122–0.8667	0.9947	-0.52452
ME_v_ on T_sa_	0.9323	1.4448	1.0982–1.8162	0.96555	0.74106

p<0.0001 for all values of r^2^.

ME_v_ = middle-ear volume; GMS = geometric mean of skull-size measurements; BC_v_ = braincase volume; T_sa_ = tympanum surface area.

*Slopes determined to be significantly different from isometry using the slope test function provided by SMATR for R (for test statistic, p<0.0001).

### Acoustic properties

**Fig 15:**
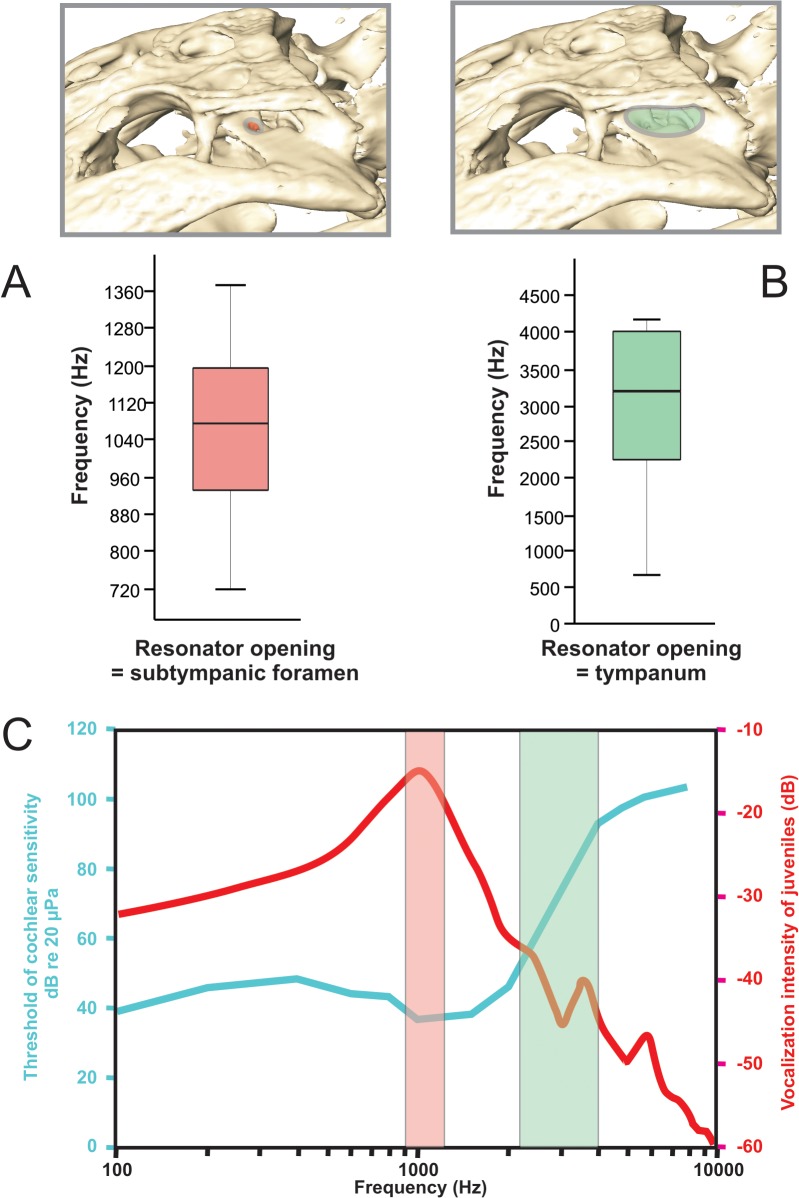
Schematic showing calculated resonant frequencies for A) subtympanic foramen (pink oval) as candidate resonator opening along with plot showing calculated resonant frequencies for an ontogenetic series of alligators. B) tympanic membrane (green oval) as candidate resonator opening along with plot showing calculated resonant frequencies for an ontogenetic series of Alligator. C) Graph adapted from Higgs et al. (2008), showing frequencies of greatest cochlear sensitivity (blue) and juvenile vocalization intensity (red) with the results of the auditory performance analysis superimposed as pink and green bars. Superimposed resonance frequencies show correlation between resonance with the subtympanic foramen to greatest cochlear sensitivity and juvenile vocalization frequencies.

Helmholtz resonance calculations for each candidate resonator opening (the tympanum and the subtympanic foramen) over the ontogenetic series returned a relatively narrow band of resonant frequencies. For the tympanic membrane, resonant frequencies between 2200 and 4000 Hz were returned (Fig 15A), whereas the subtympanic foramen returned resonant frequencies for the growth series ranging between 920 and 1200 Hz (Fig 15B). These results indicate a middle ear that is responsive to a rather restricted range of frequencies throughout ontogeny. More compelling is the fact that one of these frequency ranges, that of the subtympanic foramen, strongly matches the experimentally measured best frequencies for both the lowest threshold of cochlear sensitivity and the greatest intensity of juvenile vocalizations (Fig 15C) [40].

## Discussion

In sauropsids generally, the middle ear is composed of a pneumatically inflated epithelial sinus that is situated lateral to the bones of the braincase, medial to the bony elements of the suspensorium, and constrained elsewhere by soft tissues (e.g., muscles). In two lineages, crocodyliforms and bird-line dinosaurs, secondary outgrowths of the middle ear become insinuated within the bones of the braincase and the bones of the suspensorium. The involvement of secondary diverticula with the braincase in the avian lineage is hypothesized to be largely a consequence of the intersection of an advancing epithelial tissue front with a relatively expanded cranial vault [21]. In crocodyliforms, on the other hand, the middle-ear sinus is entrapped by a suspensorium that becomes increasingly robust and ultimately sutured to the lateral wall of the braincase, making secondary diverticular excavation necessary for the expanding middle ear. Whether it is a consequence of the expansion of the cranial vault or entrapment of the middle ear by the suspensorium, the intersection of the expanding middle-ear sinus with surrounding skeletal elements results in secondary pneumatization. Mechanistically, this secondary pneumatization is affected by the induction of osteoclast activity in the surrounding skeletal elements by signaling factors (i.e. osteopontin) produced by the pneumatic epithelia [41].

### Secondary pneumatization

The tendency toward secondary pneumatization in crocodylians is not a new observation, having been described by van Beneden [4] who recognized “the formation of secondary diverticula from the cavity of the middle ear” and characterized these diverticula as discrete cul-de-sacs derived from the middle-ear sinus. Additionally, van Beneden [4] predicted that conjunctions between adjacent diverticula would result in the opening of communicating passages. Work documenting the embryonic development of the middle ear in *Alligator mississippiensis* [6] and *Crocodylus cataphractus* [10] demonstrated the presence of some of the secondary diverticula in late-stage embryos. Simonetta [6] figured the presence of the basioccipital and pterygoid diverticula of the pharyngotympanic sinus in an alligator embryo with a head length of 36 mm, but these were not documented as discrete diverticula. Müller [10] described three phases of sinus development: (1) an early embryonic phase in which the “intertympanales” system (median pharyngeal system) is developed; (2) a late embryonic phase in which the quadrate, laterosphenoid (= prootic), supraoccipital (= intertympanic), basioccipital, and exoccipital (= otoccipital) diverticula are present; and (3) a post-embryonic phase in which the communication between the basioccipital diverticula and the “intertympanales” system is established (described therein as the “canalis posterior”).

In the present study, the youngest specimens are at the final days of embryonic development (Ferguson Stage 25, i.e., Day 51–60) [42], and at this point exhibit nearly the complete suite of secondary diverticula. Every element of the chondrocranium and splanchnocranium (except the squamosal) is pneumatized, and only the parietal diverticulum (dermatocranium) is lacking. We also observe that in late-stage embryos a large degree of diverticular confluence has already occurred, such as the intertympanic communication through the supraoccipital, as well as the communication between the basisphenoid diverticulum of the median pharyngeal sinus and the recessus epitubaricum of the pharyngotympanic sinus. Post-embryonically, interdiverticular confluences that were incomplete or incipient in embryos are completed, such as the communication between the antrum of the median pharyngeal sinus and the basioccipital diverticulum of the pharyngotympanic sinus, or the communications between the otoccipital and intertympanic diverticula. This pneumatic expansion, including the invasion of the dermatocranium (parietal diverticulum) and the increasing confluence between diverticula continues until about 100 mm DSL. Thereafter, the conformation of the middle-ear pneumatic system is relatively static, except that in adult individuals (> 300 mm DSL) there is a trend toward the reduction of more peripherally located diverticula (e.g., the pterygoid diverticulum, the laterosphenoid expansion of the prootic diverticulum, and the articular diverticulum and its communicating siphonium).

The diverticular infiltrations of the basicranium (i.e., the basisphenoid and basioccipital diverticula) are often depicted schematically as elongate tubes communicating with the median pharyngeal sinus [5,7,9]. Alternatively, these diverticula are demonstrated in parasagittal section [4,14,39], which tends to present an oblique transect through these spaces. The result is the appearance of a dorsoventrally elongated passage. Verticalization of the basicranium is a phenomenon that occurs during ontogeny in crocodylians and has a demonstrable effect on the angles of the jaw adductor muscles [14] (see Brochu [43] and Gold et al. [44] for a phylogenetic perspective on verticalization in crocodylian evolution). The verticalization of the basicranium also affects the pharyngotympanic and median pharyngeal tubes, clearly causing them to become vertically elongated. In very mature alligators, the conformation of the basisphenoid and basioccipital diverticula is not greatly altered by the verticalization of the braincase (Fig 13). Very mature individuals of other taxa, such as *Tomistoma* (USNM 211323), however, show a marked tendency toward narrowing and vertical elongation of the basisphenoid and basioccipital diverticula and especially the communicating passages between these diverticula and the median pharyngeal sinus.

### Constraints on diverticular expansion

The rapid and extensive inflation of the secondary diverticula, which expand into every element of the neurocranium and much of the suspensorium by the time of hatching, suggests an adventitious growth pattern consistent with Witmer’s [45] “epithelial hypothesis,” which regards pneumaticity ultimately as an opportunistic process whereby diverticula expand within the constraints imposed by other structures (e.g., muscles, eyeball, blood vessels, nerves, bones) and biomechanical integrity. There are particular structural constraints that are evident in the manner in which the diverticula expand. For example, pillars of bone stand where diverticula transect sutural boundaries, such as the pillar at the otoccipital-supraoccipital suture that remains after the otoccipital and intertympanic diverticula become confluent. Although it may seem that this pillar exists to maintain the integrity of the sutural contact, it should be noted that this particular pillar overlies a neurosensory structure, the apex of the caudal semicircular canal of the inner ear. Accordingly, neurosensory and neurovascular structures are the primary constraining structures shaping the advance of epithelial diverticula. Similar to the case with the caudal semicircular canal, the resorptive expansion of the epithelia of the parietal diverticula is inhibited by tissues surrounding the apex of the rostral semicircular canal, resulting in a pillar of bone at the suture between the parietal and supraoccipital where the parietal diverticulum conjoins the pharyngotympanic sinus. Many neural structures, such as the palatine and hyomandibular branches of the seventh cranial (facial) nerve, have pathways that follow the external contours of the braincase elements, and as such are peripheral to the epithelial sinuses and diverticula. Other neurovascular structures such as the trigeminal ganglion, the carotid artery, and cranial nerves IX–XI collectively, have trajectories or topologies that place them in the path of advancing epithelial fronts. In these instances, the inflating diverticulum is either deflected by the neurovascular structure (e.g., the prootic diverticulum is constrained to pass dorsal to the trigeminal ganglion and medial to the tympanic branch of the trigeminal nerve) or the neurovascular structure is encircled by the diverticulum. Other bony remnants ostensibly are not representative of a constraint upon diverticular expansion, such as the parasagittal pillars between the parietal and supraoccipital bones. These are merely spandrels, both in the structural sense and the adaptive sense of Gould and Lewontin [46], representing pillars at the intersection of two diverticula, in this case the intersection of the paired parietal diverticula.

### Interpretations of auditory function

We interpret our finding that the middle-ear volume is in isometry with the tympanic membrane surface area as a functional constraint reflecting the need to preserve auditory function during ontogeny. We also note that resonant frequencies of the middle-ear sinus system have the potential to inform us about sound frequencies that are biologically or ecologically relevant. In our analysis we have discovered a compelling match between the resonant frequency of sound energy conducted through the subtympanic foramen and both the frequencies of best cochlear sensitivity and greatest vocalization intensity of juveniles (Fig 15), suggesting that acoustic resonance could be a factor determining the extent if not the form of the pneumatic sinuses. The behavioral response of adult crocodylians to juvenile distress calls has been well documented [47–49], and the use of juvenile distress calls is widely distributed among extant crocodylians [50,51]. Likewise, the subtympanic foramen is a feature common to the middle ear of all extant crocodylians (and also many extinct taxa within the clade Mesoeucrocodylia). That the resonant properties of the middle ear remain remarkably constant during ontogeny is in contrast to the recent discovery that vocal tract resonance produces formant frequencies as a function of size [52]. Thus far we have only documented correlations of auditory resonance with cochlear sensitivity and juvenile vocalization frequencies in *Alligator mississippiensis*, and further analyses are needed to see if the correlation holds for other extant crocodylian taxa.

### Ontogeny as a predictive tool for phylogenetic studies

An understanding of the identity, topology, and developmental sequence for the sinuses and discrete diverticula that excavate the skull of the alligator can be of great utility. Coupled with knowledge of the intersections and confluences that develop between these pneumatic structures ontogenetically, we have the potential to develop predictive tests to assess the homology of purported pneumatic features in fossil taxa as identified by diagnostic osteological correlates. Additionally, our hypotheses of constraints influencing diverticular expansion, and hypotheses of exapted functional roles and their potentially correlated behaviors can also be placed in a phylogenetic framework. Our preliminary investigations [20,21] have already demonstrated the recapitulation of diverticular development in the phylogenetic trajectory of crocodylomorphs (e.g., the beginnings of diverticular infiltrations are seen in basal crocodylomorphs, and diverticular patterning in basal Mesoeucrocodylia approximates the pattern seen early in ontogeny in crown Crocodylia), and the response of fossil taxa occupying similar ecological niches to similar constraints on diverticular expansion (e.g., feeding specialists with attenuated snouts and enlarged adductor chambers, such as the thalattosuchian *Pelagosaurus* and the extant crocodylian *Gavialis*, show a reduction in the number, extent, and communications between diverticula, whereas feeding generalists, such as the oreinirostral sebecid *Hamadasuchus* and the extant crocodylian *Paleosuchus*, have greatly expanded diverticula).

## Supporting Information

S1 File 3D pdf of perinatal Alligator mississippiensis (OUVC 10606). Using Adobe Acrobat (or the free Reader), click on figure to activate 3D functionality. Skeletal elements, soft-tissue endocasts, and paratympanic sinus and diverticula can be manipulated, selected, and ‘turned-off’ and ‘turned-on’ individually or as groups.
(PDF)
