# Supplementary figures and images for: Ontogeny of the Middle-Ear Air-Sinus System in Alligator mississippiensis (Archosauria: Crocodylia)
Source: PLoS One. 2015 Sep 23;10(9):e0137060. doi: 10.1371/journal.pone.0137060 (PMC4580574; doi:10.1371/journal.pone.0137060)

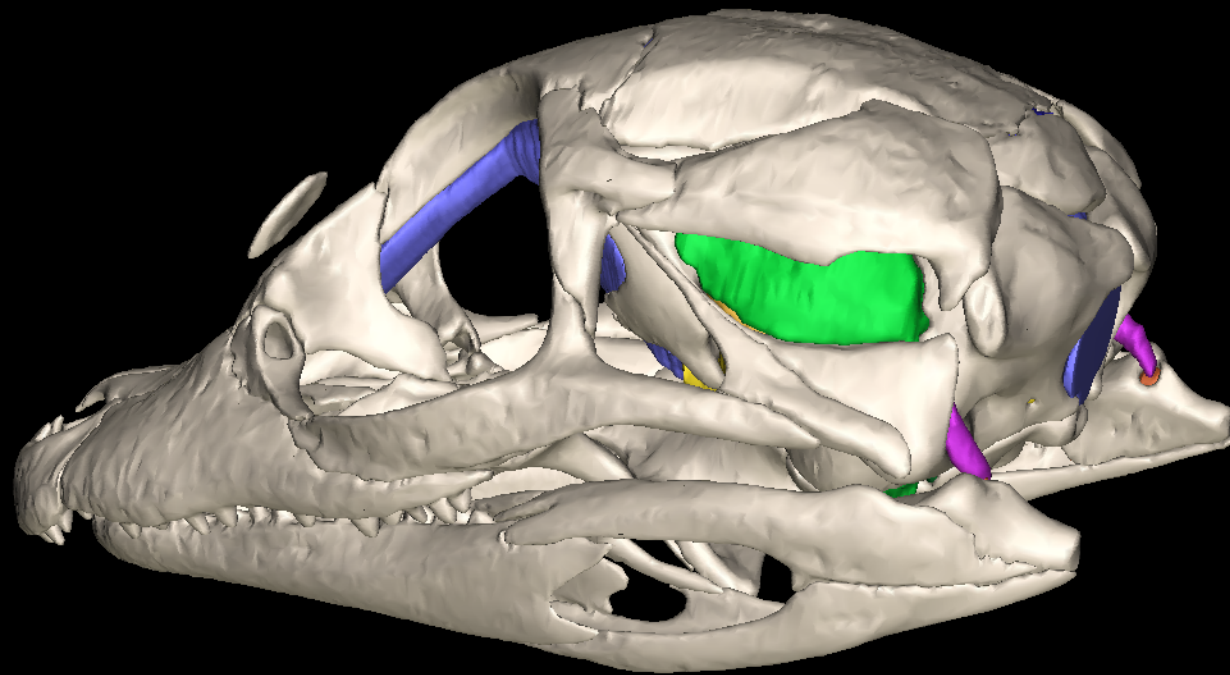

Supplement: S1 File — Using Adobe Acrobat (or the free Reader), click on figure to activate 3D functionality. Skeletal elements, soft-tissue endocasts, and paratympanic sinus and diverticula can be manipulated, selected, and ‘turned-off’ and ‘turned-on’ individually or as groups. (PDF) [file pone.0137060.s001.pdf]
